# Supplementary material for: Accumulation of storage proteins in plant seeds is mediated by amyloid formation
Source: PLoS Biol. 2020 Jul 23;18(7):e3000564. doi: 10.1371/journal.pbio.3000564 (PMC7377382; doi:10.1371/journal.pbio.3000564)
Supplement: S1 Fig — Amino acid sequence of Vicilin is shown. Peptides identified by mass spectrometry are indicated in red. Signal peptide is highlighted in green. (PDF) [file pbio.3000564.s001.pdf]

Vicilin 47k [*Pisum sativum*] CBK38922.1

MH+ (mono): 1.008   MH+ (avg): 1.008   Tolerance (Da): 0.700   Number of Peaks: 1012

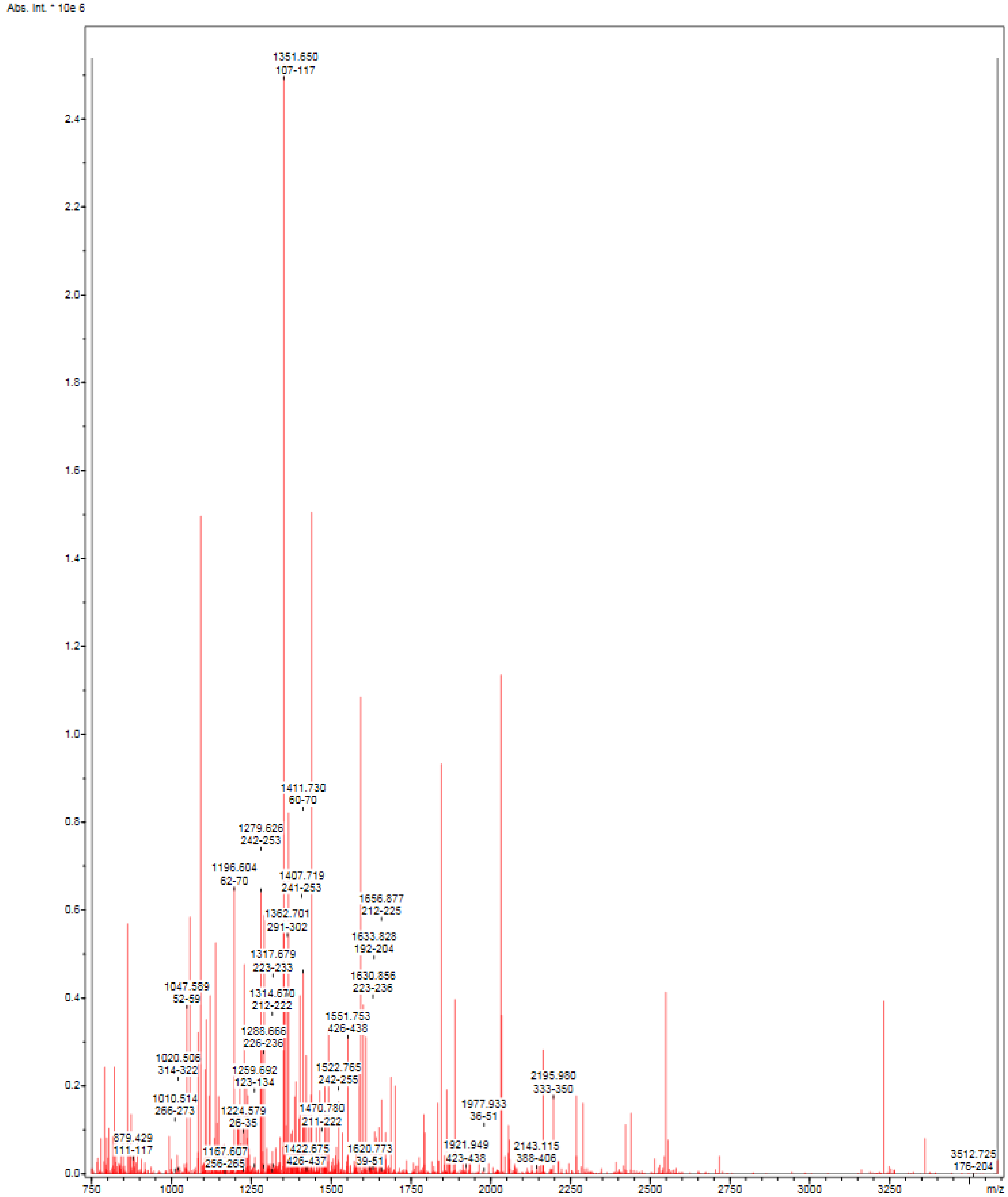

MAATPIKPLMLLAIAFLASVCVSSRSDQENPFIFKSNRFQTLYENENGHIRLLQKFDKRSKIFENLQNYRLL
EYKSKPHTLFLPQYTDADFILVVLNGKATLTVLKSNDRNSFNLERGDTIKLPAGTIAYLANRDDNEDLRVLD
LAIPVNKPGQLQSFLLSGTQNQPSLLSGFSKNILEAAFNTNYEEIEKVLLEQQEQEPQHRRSLKDRRQEIN
EENVIVKVSREQIEELSKNAKSSSKKSVSSESGPFNLRSRNPIYSNKFGKFFEITPEKNQQLQDLDIFVNSV
DIKEGSLLLPNYNSRAIVIVTVTEGKGDFELVGQRNENQGKENDKEEEQEEETSKQVQLYKAKLSPGDVF
VIPAGHPVAINASSDLNLIGFGINAENNERNFLAGEEDNVISQVQRPVKELAFPGSSHEIDRLLKNQKQSYF
ANAQPLQRE

S1 Fig. Mass spectrometry identification data of the *P. sativum* Vicilin (47 kDa). Amino acid sequence of Vicilin is shown. Peptides identified by mass spectrometry are indicated in red. Signal peptide is shown in green.
