# Supplementary figures and images for: Accumulation of storage proteins in plant seeds is mediated by amyloid formation
Source: PLoS Biol. 2020 Jul 23;18(7):e3000564. doi: 10.1371/journal.pbio.3000564 (PMC7377382; doi:10.1371/journal.pbio.3000564)

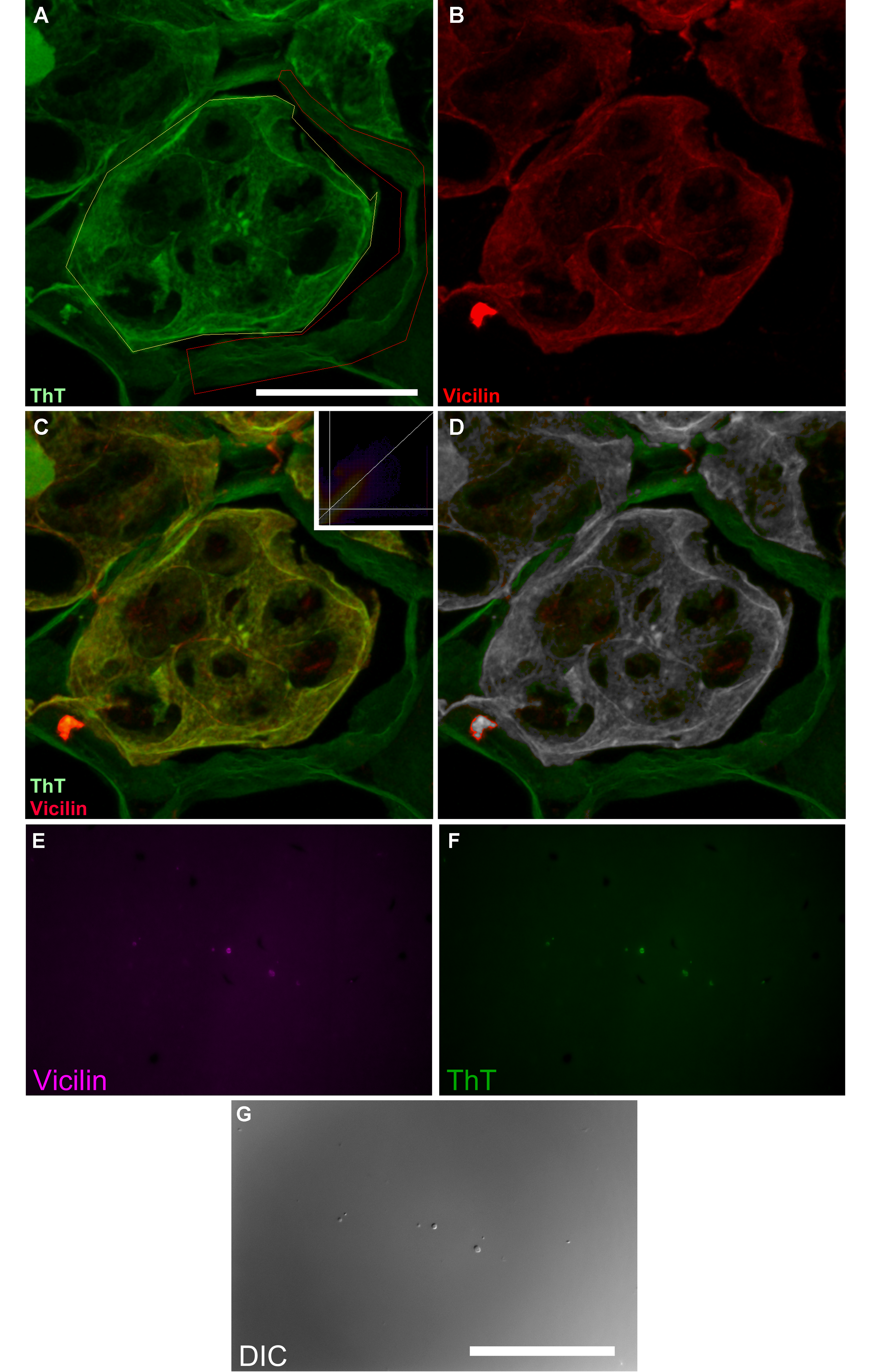

Supplement: S2 Fig — (A–D) Laser scanning confocal microscopy of the in situ hybridization of anti-Vicilin antibodies with ThT on pea seed cryosections is shown. ThT channel of fluorescence is green, anti-Vicilin antibody—red, overlay—yellow and gray. (A) ThT channel, yellow outline shows the region used for colocalization analysis of cytoplasm; (B) anti-Vicilin; (C) overlay of the anti-Vicilin and ThT channels, insert shows correlation diagram between channels; (D) anti-Vicilin and ThT channels, colocalized pixels shown in grayscale. (E–G) ThT staining of PB isolated from pea seeds. (E) In situ hybridization with anti-Vicilin antibodies. (F) ThT staining of the same slide. (G) Differential interference contrast. Scale bar is equal to 50 μm. PB, protein bodies; ThT, Thioflavin T. (TIF) [file pbio.3000564.s002.tif]

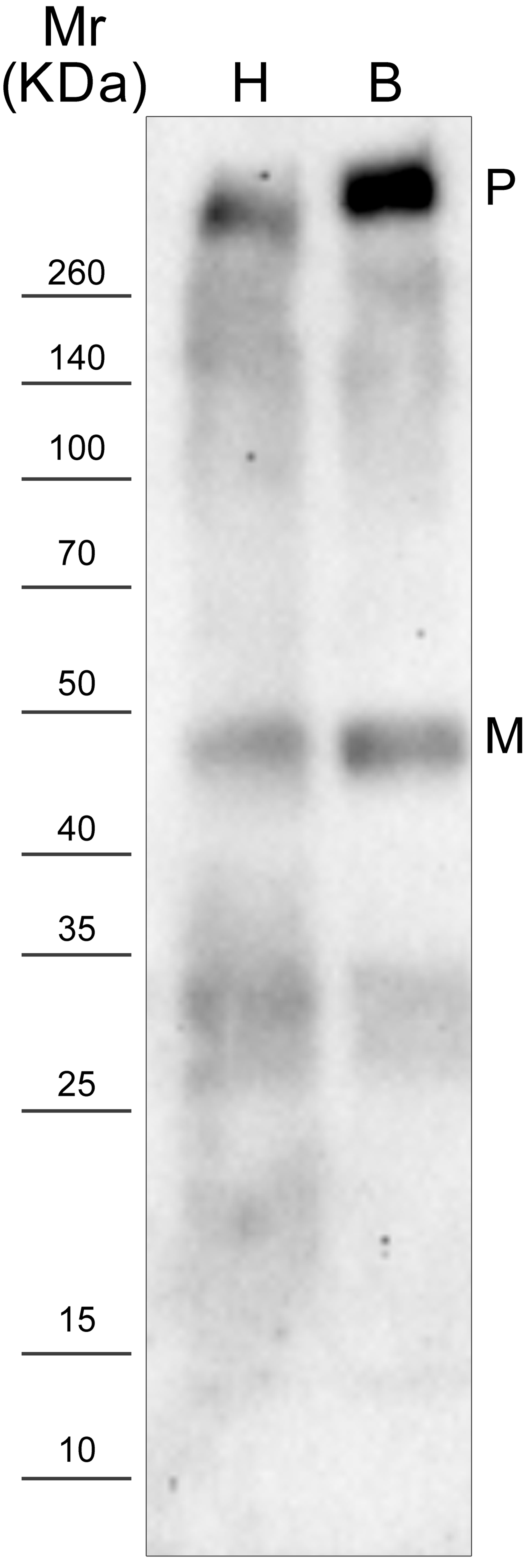

Supplement: S3 Fig — Western blot image is shown, polyclonal anti-Vicilin antibody was used. B: canned peas produced by Bonduelle (Bonduelle Group, France), H: the same by Heinz (H.J. Heinz, Pittsburgh, PA), P: polymers, M: monomers. All samples were loaded onto the gel after 5 min boiling in buffer with 2% SDS. Corresponding molecular weights are shown (kDa). SDS, Sodium Dodecyl Sulfate. (TIF) [file pbio.3000564.s003.tif]

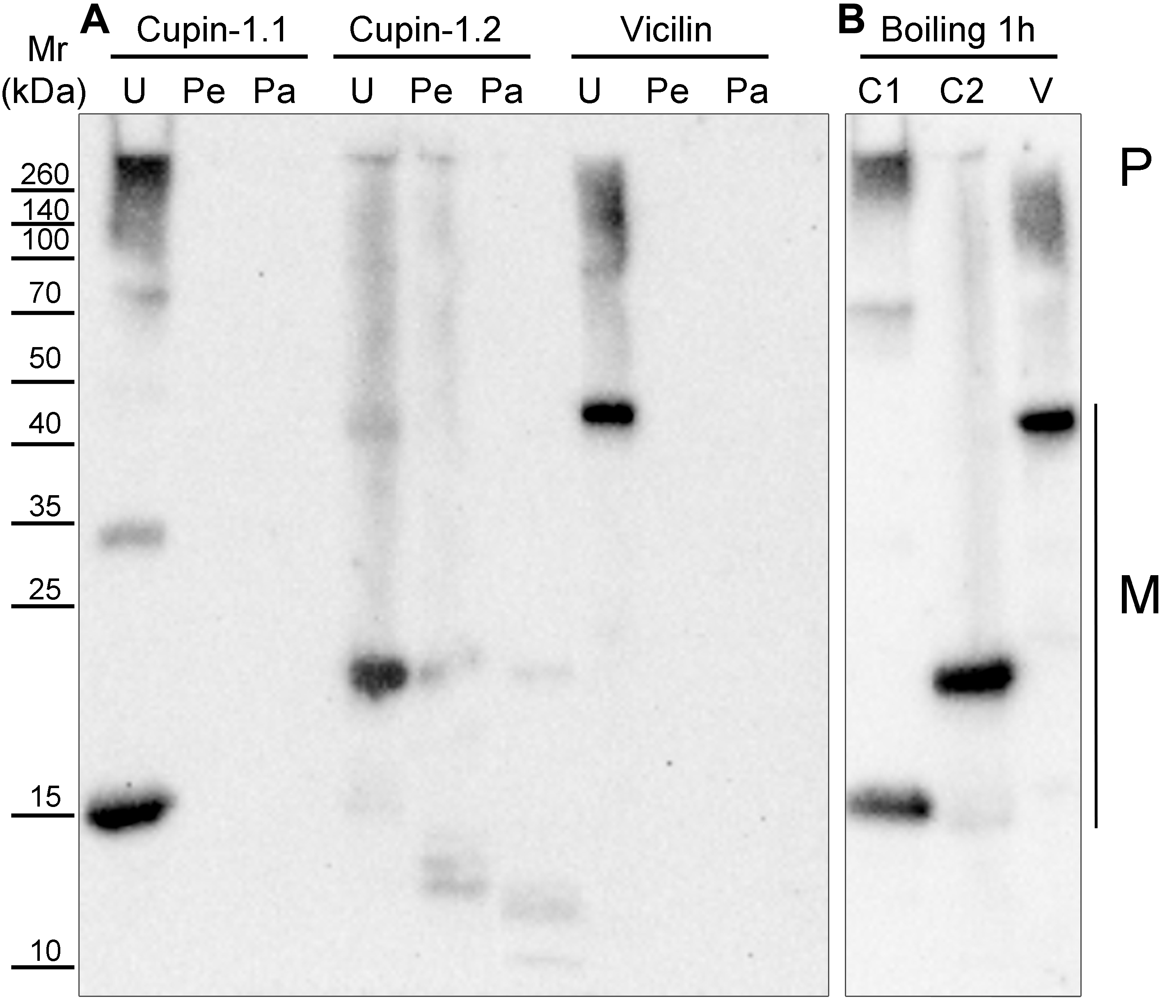

Supplement: S4 Fig — Western blot image is shown, polyclonal anti-Vicilin antibody was used. (A) Protease resistance of the Vicilin, Cupin-1.1, and Cupin-1.2 in vitro obtained fibrils. U, untreated samples; Pe, treated with pepsin; Pa, treated with pepsin and pancreatin; P, polymers; M, monomers. All samples in sections g–h were loaded onto the gel after 5 min boiling in buffer with 2% SDS. Corresponding molecular weights are shown (kDa). (B) Vicilin (V), Cupin-1.1 (C1), and Cupin-1.2 (C2) fibrils obtained in vitro were boiled for 1 h in buffer contained 2% SDS and then loaded onto the gel. Corresponding molecular weights are shown (kDa). SDS, Sodium Dodecyl Sulfate. (TIF) [file pbio.3000564.s004.tif]

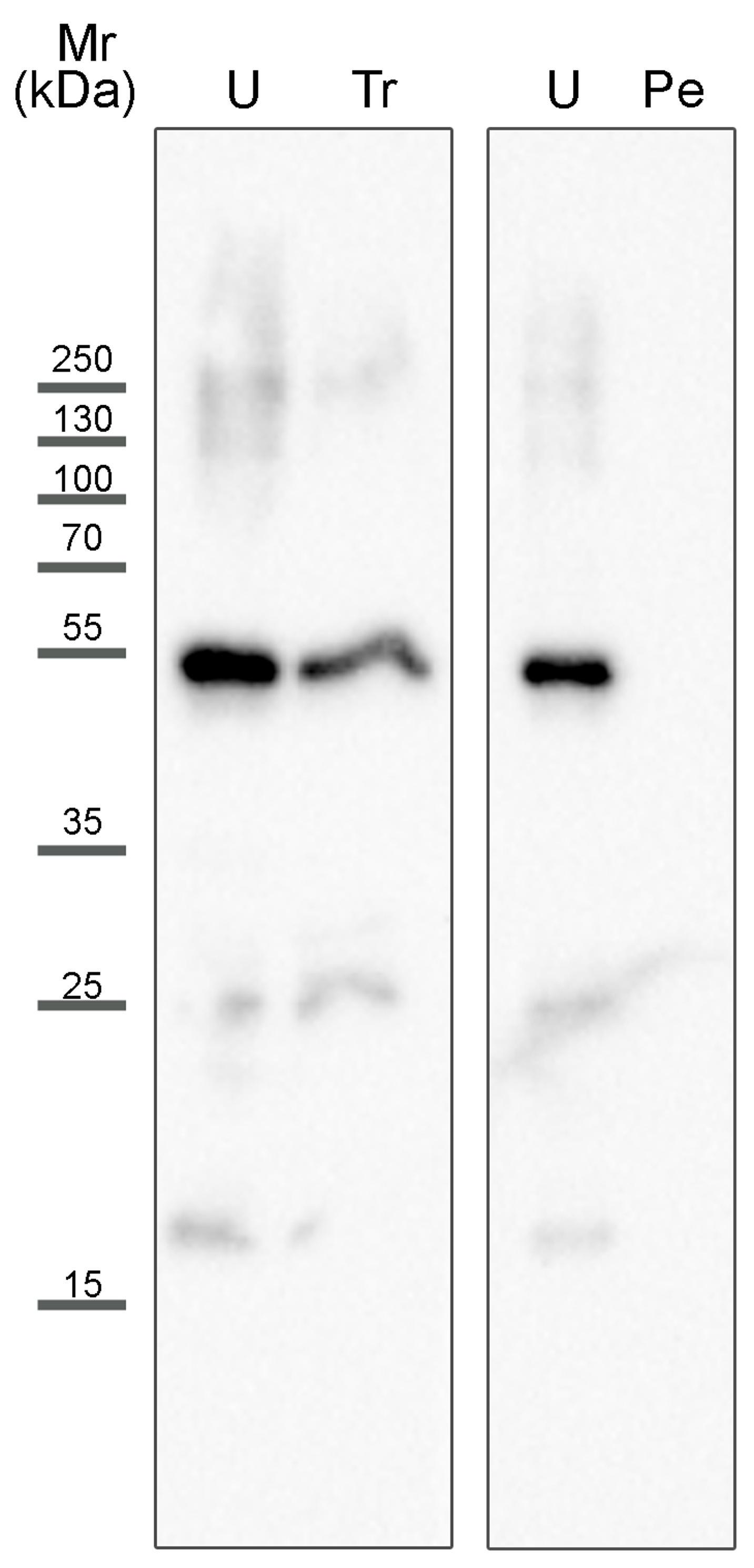

Supplement: S5 Fig — Western blot image is shown; polyclonal anti-Vicilin antibody was used. U, untreated samples; Tr, treated with trypsin at 1:60 trypsin-to-total protein mass ratio for 45 min at 37°C; Pe, treated with pepsin as described in “Materials and methods.” Samples were loaded onto the gel after 5 min boiling in buffer with 2% SDS. Corresponding molecular weights are shown (kDa). SDS, Sodium Dodecyl Sulfate. (TIF) [file pbio.3000564.s005.tif]

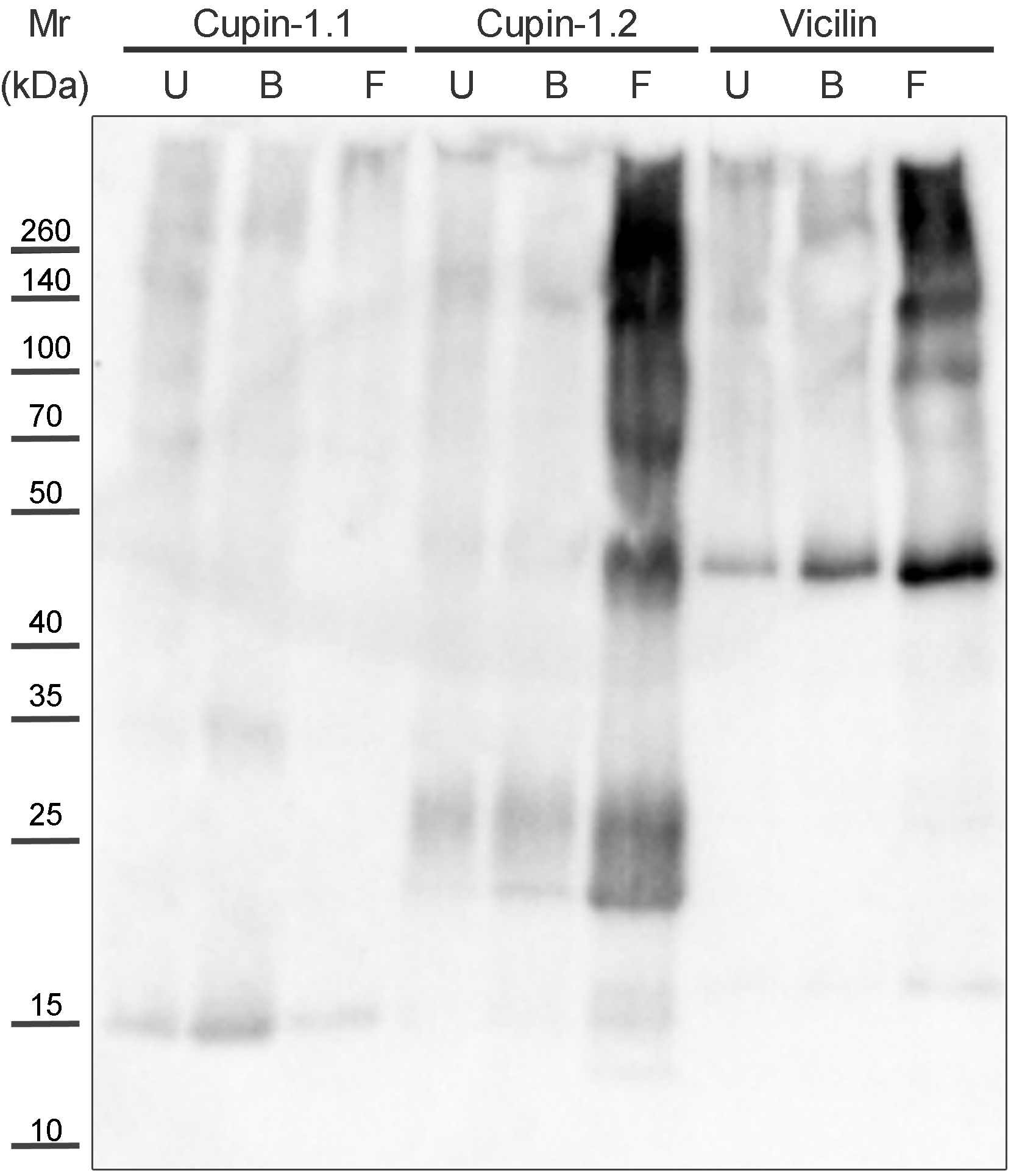

Supplement: S6 Fig — Western blot image is shown, polyclonal anti-Vicilin antibody was used. U, samples treated with cold 2% SDS; B, samples boiled in buffer containing 2% SDS; F, lyophilized samples were treated with 90% formic acid (Sigma-Aldrich, St. Louis, MO), lyophilized again, solubilized in the same volume of PBS with addition of SDS sample buffer containing 2% SDS (final concentration) and loaded onto the gel. Corresponding molecular weights are shown (kDa). SDS, Sodium Dodecyl Sulfate. (TIF) [file pbio.3000564.s006.tif]

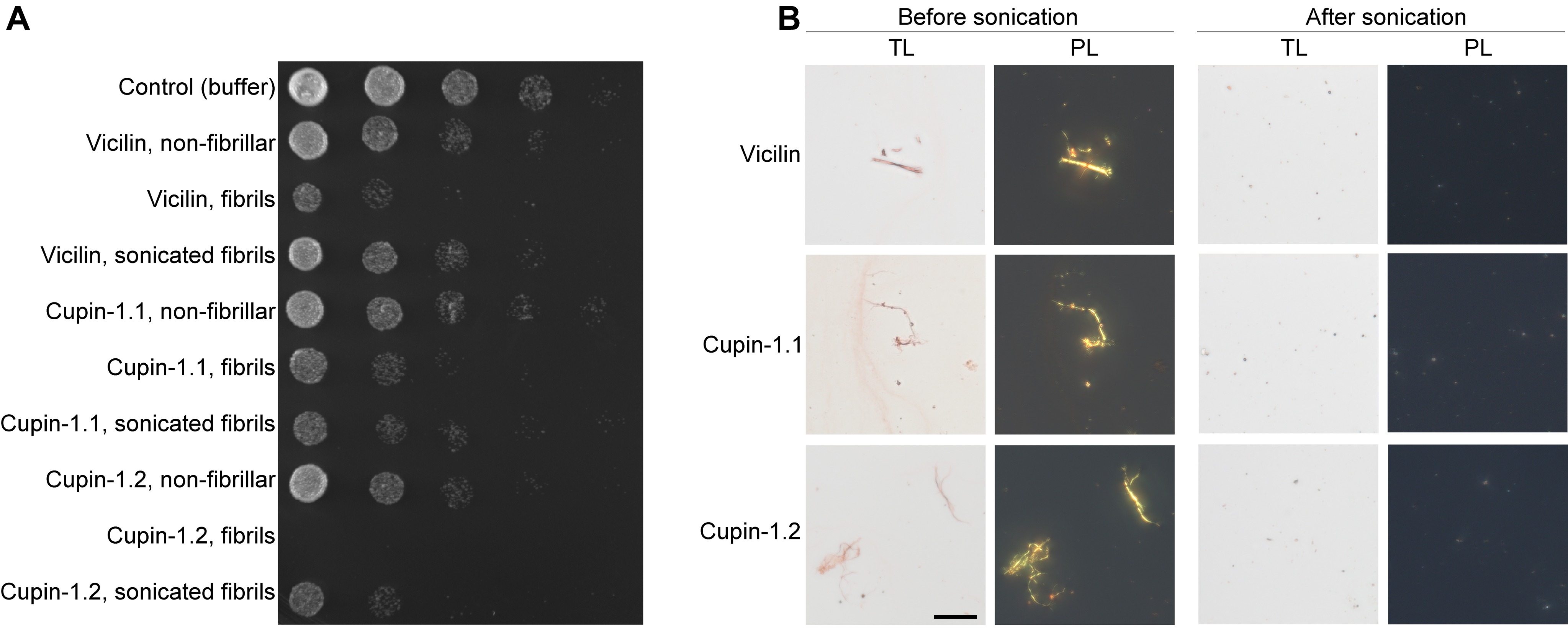

Supplement: S7 Fig — (A) A series of 10-fold dilutions of the liquid yeast culture treated with fibrillar, nonfibrillar, and disrupted by sonication aggregates of the corresponding proteins is shown. 1 mg/mL protein concentrations were used. Sonicated samples were prepared from fibrils using the following conditions: 40 s, at 60% power, Q125 sonicator (Qsonica, Newtown, CT). Picture was taken after 48 h of incubation at 30°C. (B) The effect of sonication on the fibrils of Vicilin, Cupin-1.1, and Cupin-1.2. The same sonication conditions as in Panel A have been used. Samples have been stained with CR. TL, transmitted light; PL, polarized light. The scale bar is equal to 20 μm. CR, Congo Red. (TIF) [file pbio.3000564.s007.tif]

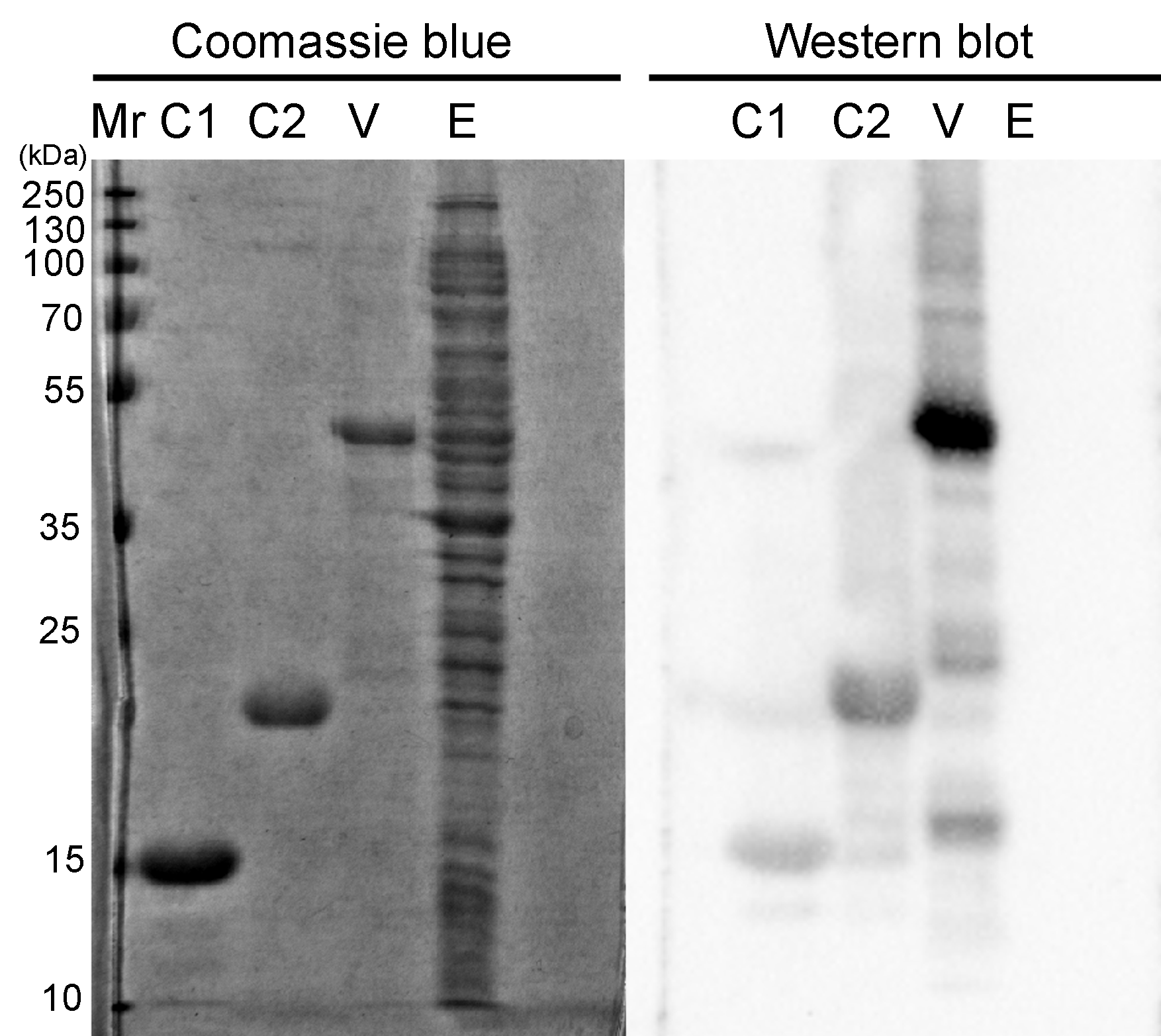

Supplement: S8 Fig — Left: Coomassie blue stained SDS-PAGE gel; right: western blot image. C1, recombinant Cupin-1.1 protein sample; C2, recombinant Cupin-1.2 sample; V, recombinant full-length Vicilin sample; E, total protein lysate of E. coli strain BL-21. All samples were boiled in buffer containing 2% SDS (final concentration) and loaded onto the gel. Corresponding molecular weights are shown (kDa). SDS, Sodium Dodecyl Sulfate. (TIF) [file pbio.3000564.s008.tif]

Fig 1C.

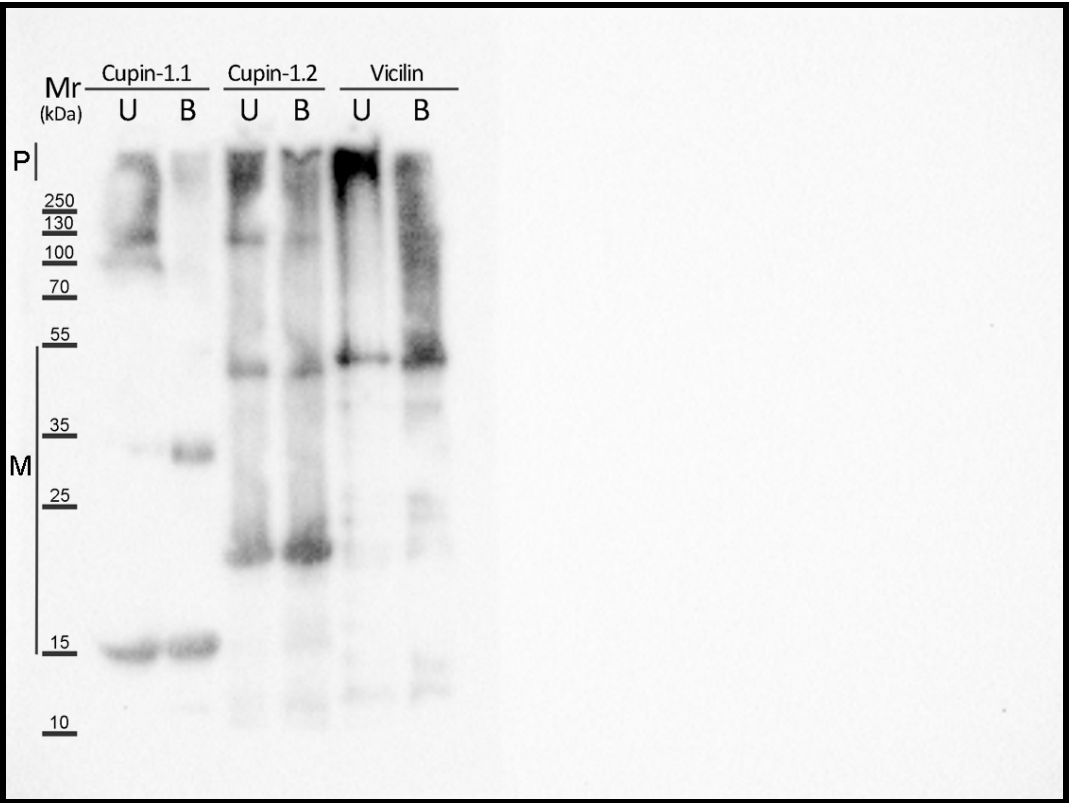

Fig 3B.

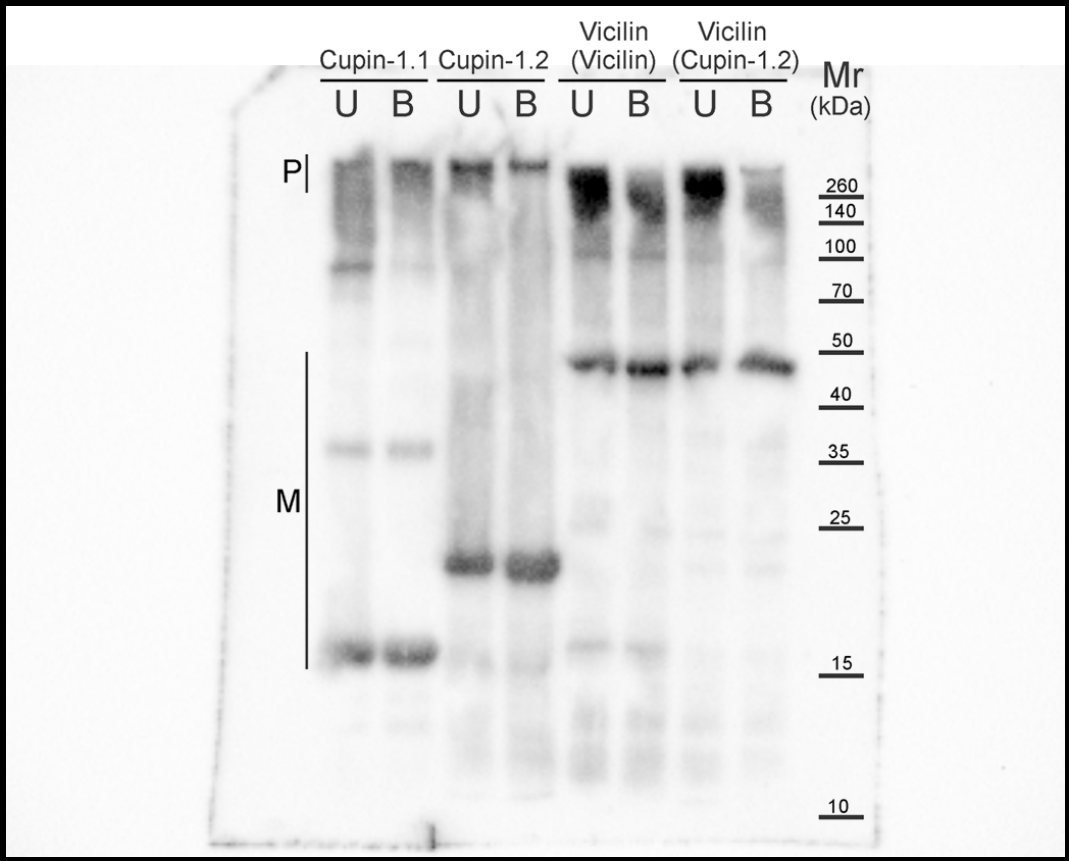

Fig 5N, left.

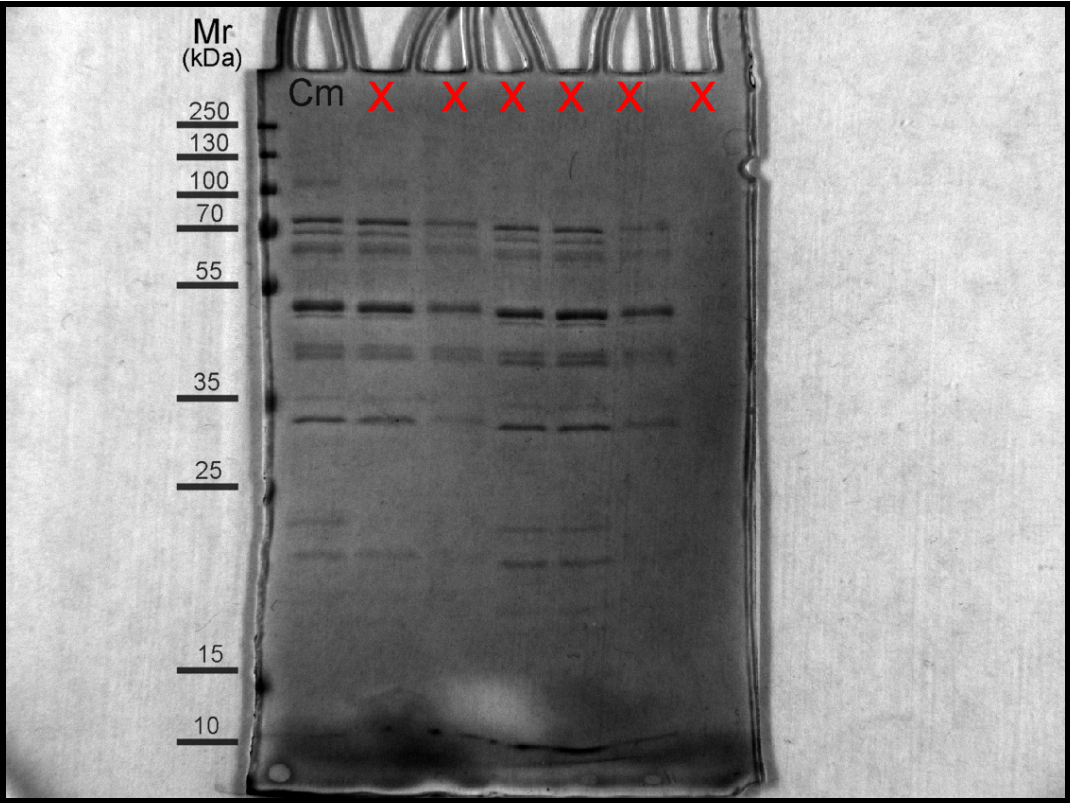

Fig 5N, right.

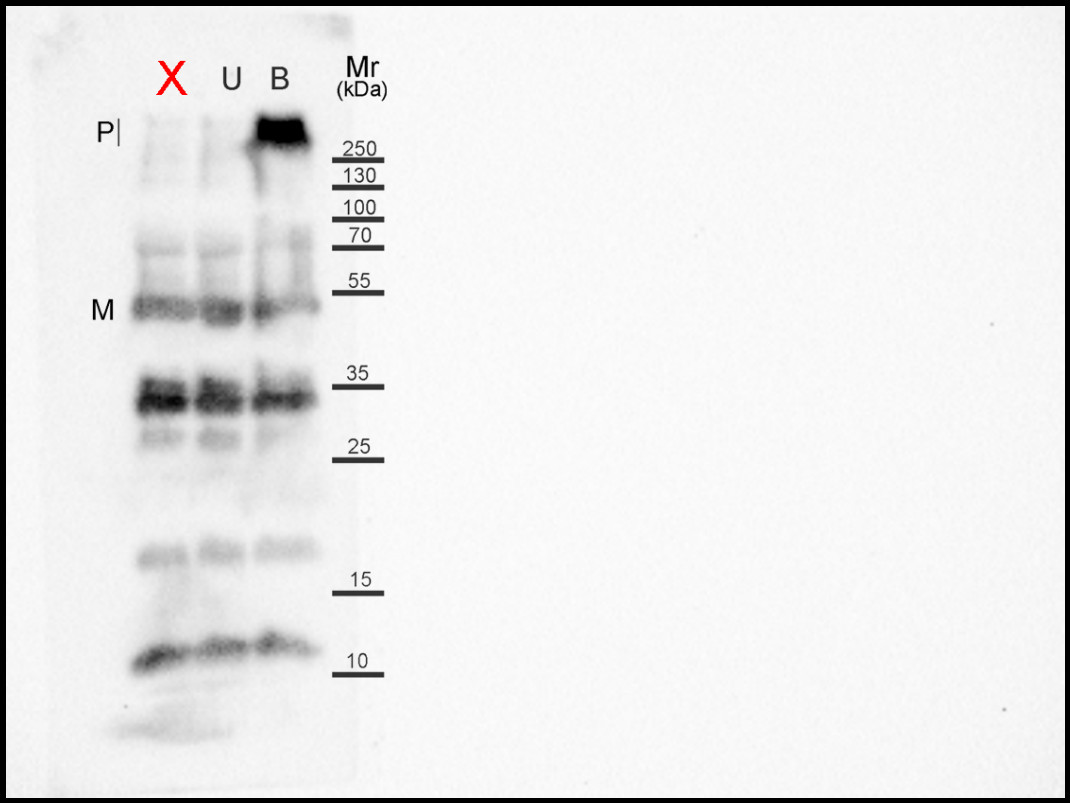

Fig 5O.

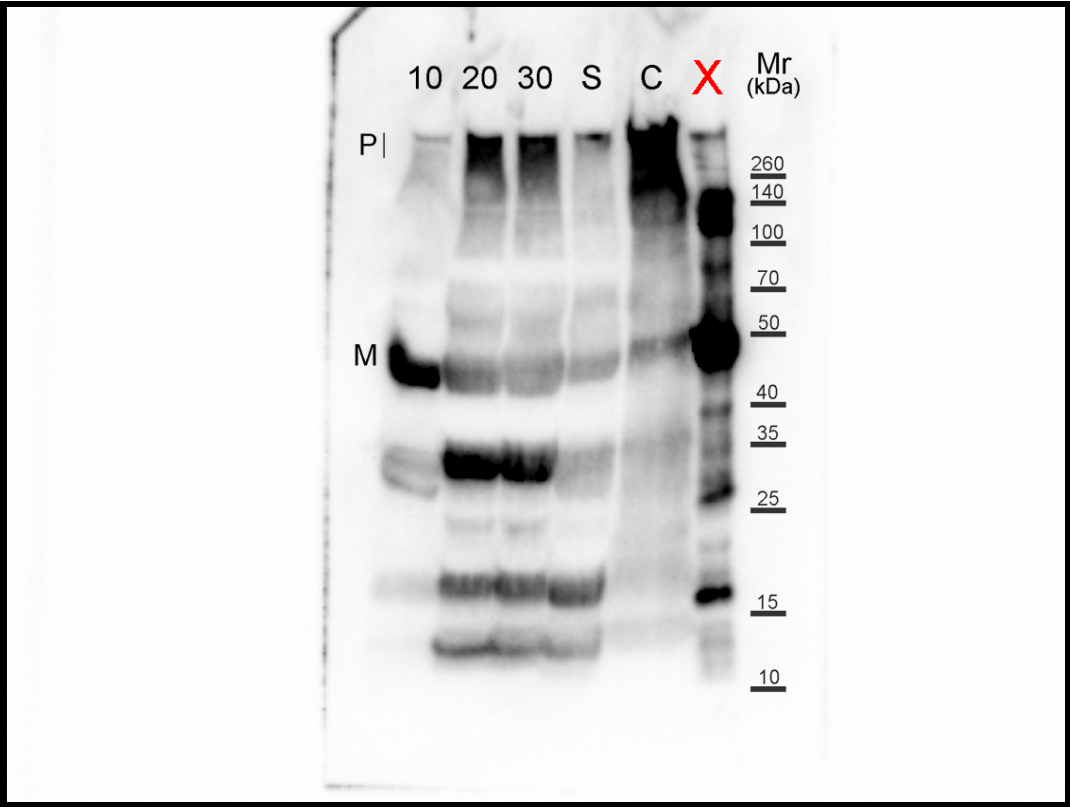

Fig 5P.

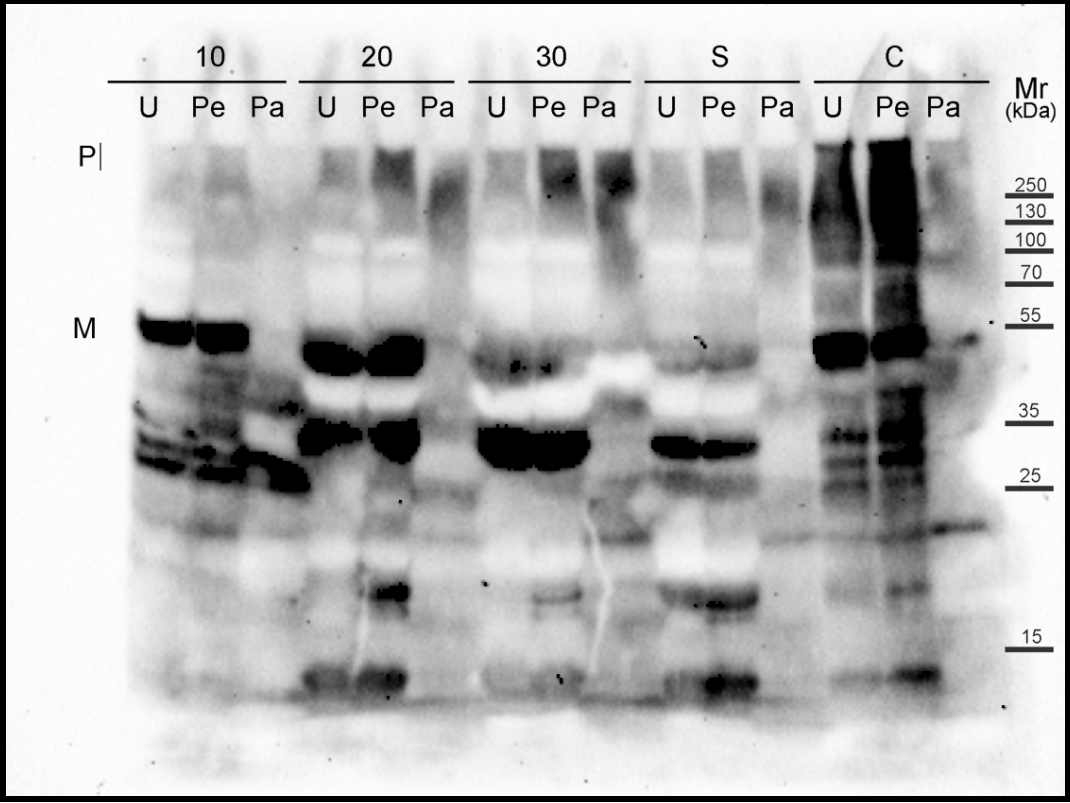

Fig S3.

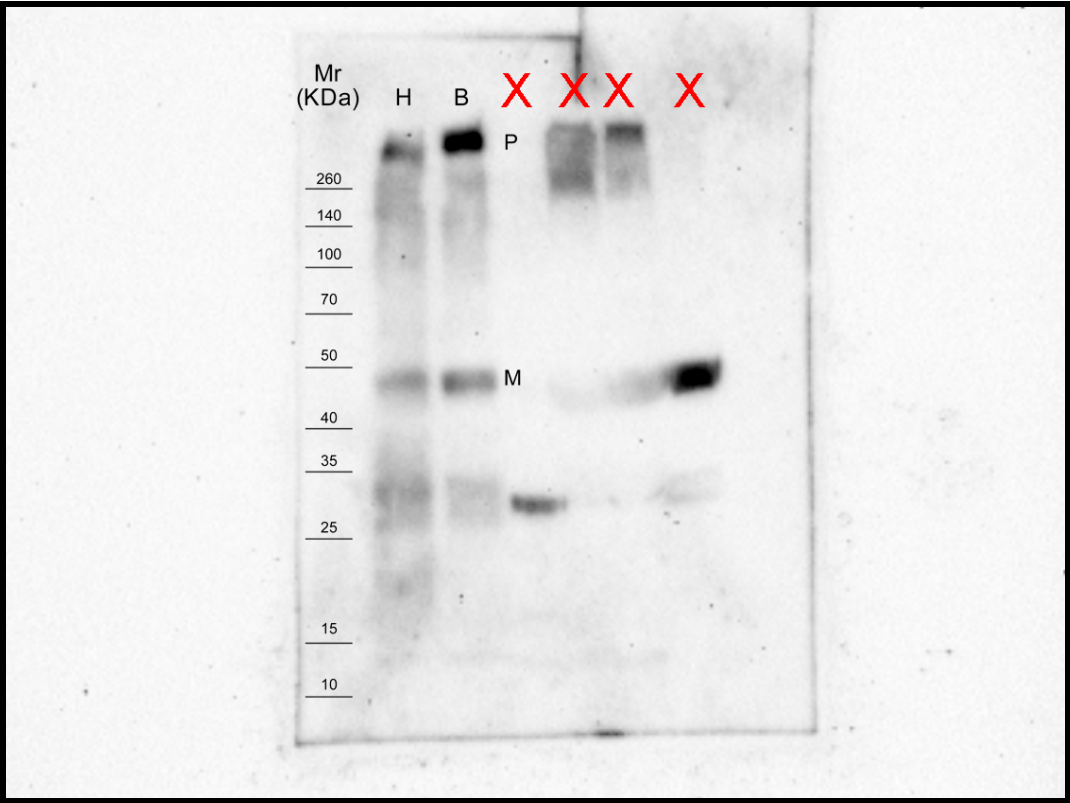

Fig S4, left.

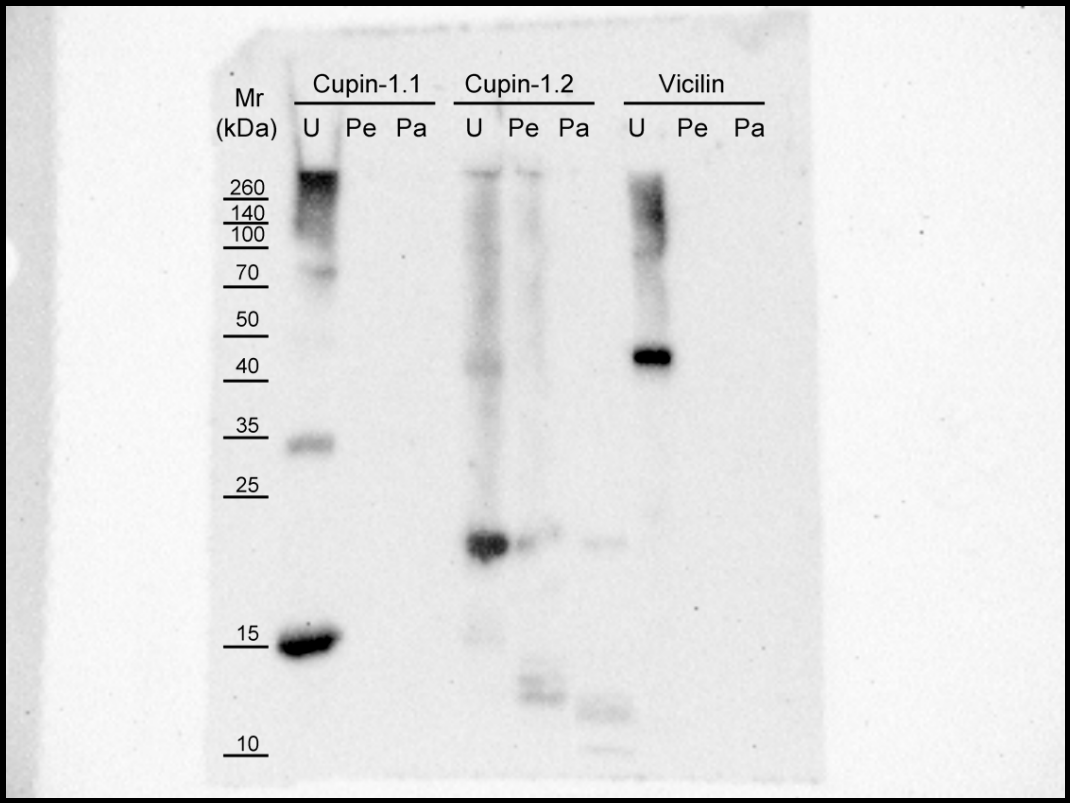

Fig S4, right.

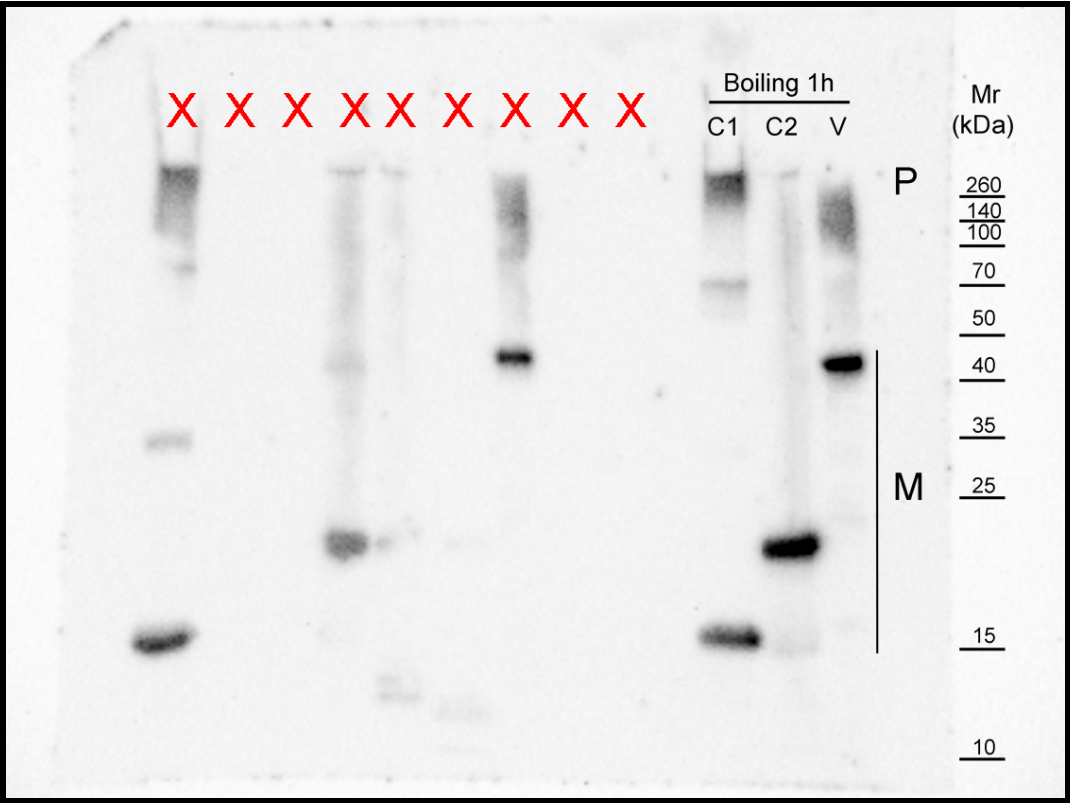

Fig S5.

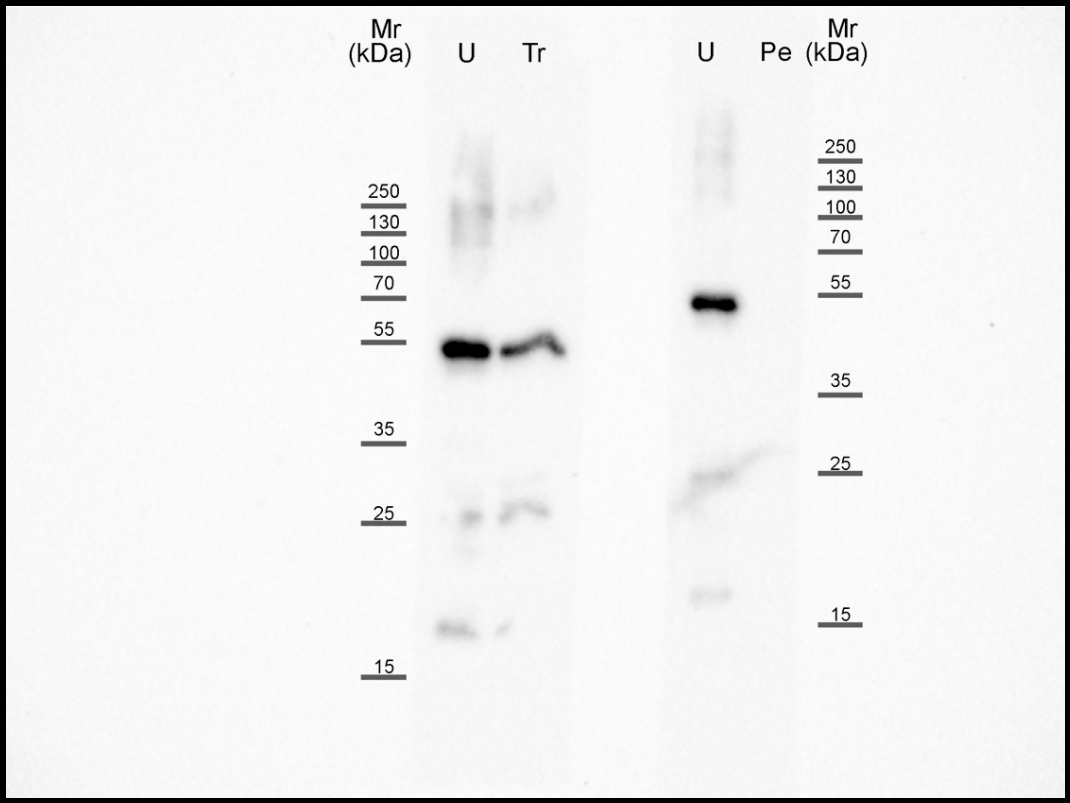

**Fig S6.**

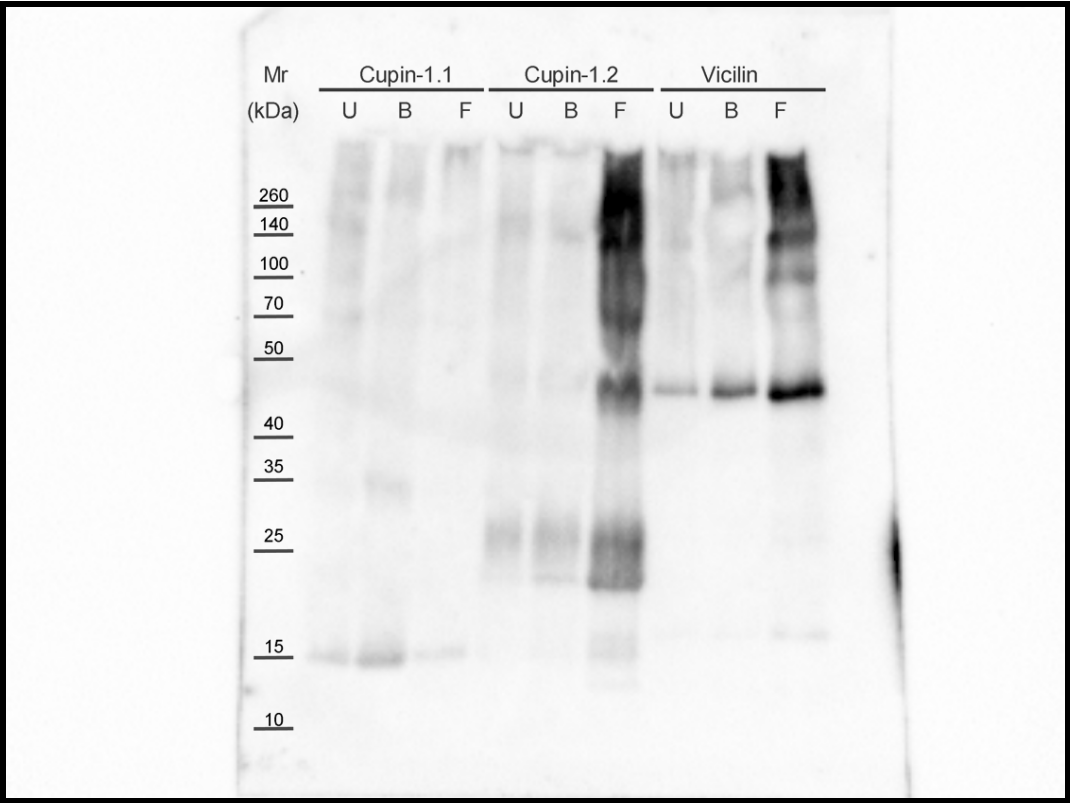

**Fig S8, left.**

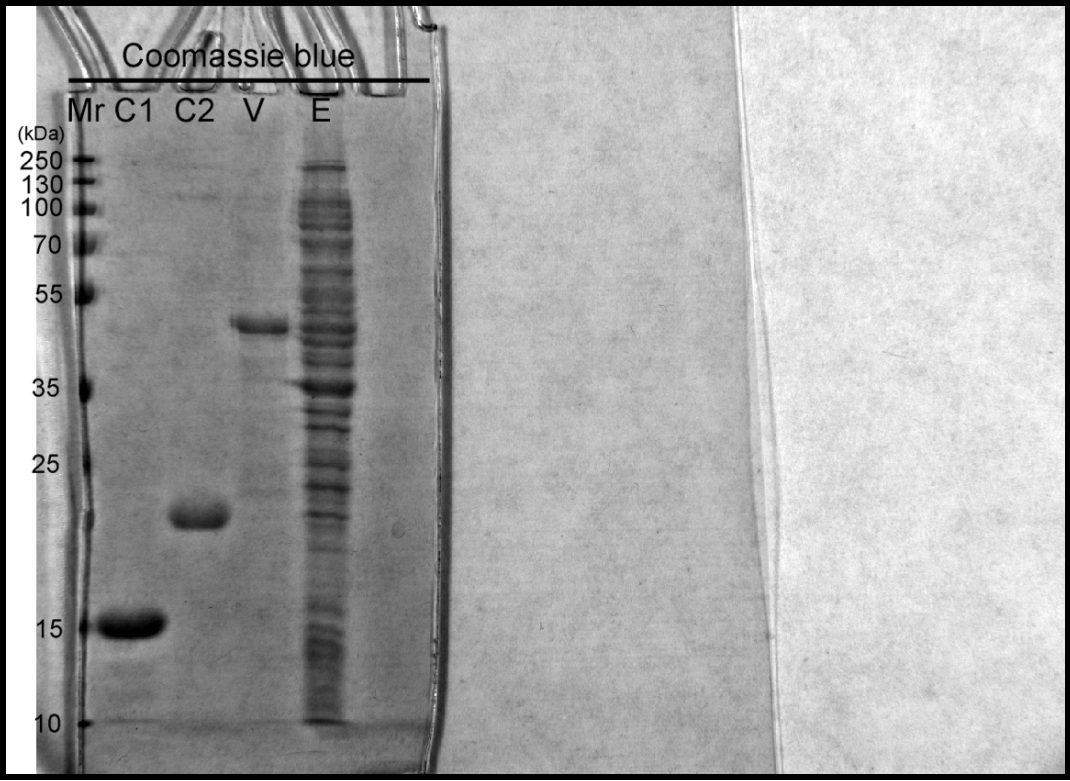

Fig S8, right.

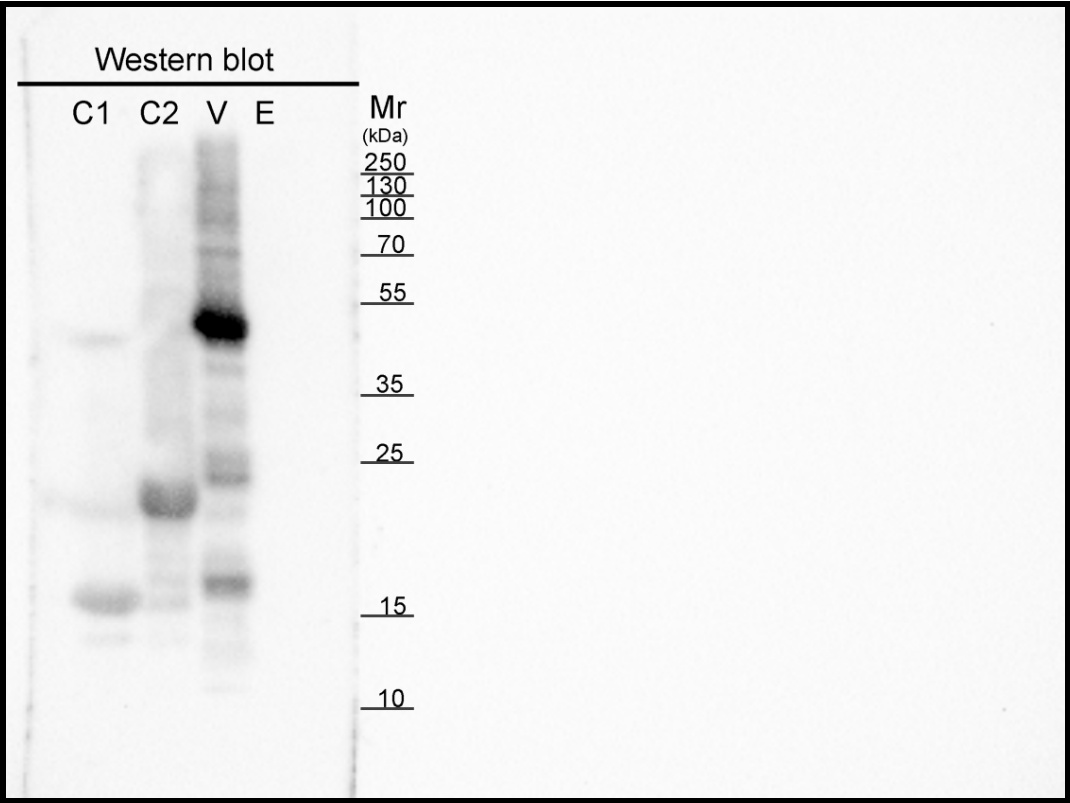

Supplement: S1 Raw Images — (PDF) [file pbio.3000564.s016.pdf]
