# Supplementary material for: Accumulation of storage proteins in plant seeds is mediated by amyloid formation
Source: PLoS Biol. 2020 Jul 23;18(7):e3000564. doi: 10.1371/journal.pbio.3000564 (PMC7377382; doi:10.1371/journal.pbio.3000564)
Supplement: S1 Table — (PDF) [file pbio.3000564.s009.pdf]

**S1 Table. Proteins identified in detergent resistant fraction of the *P. sativum* L. seeds.**

| Protein | Accession[1] | Score[2] | MW[3] | pI[4] | SC[5] | Peptides[6] |
|---|---|---|---|---|---|---|
| Vicilin | P13918.2 | 2246.41 | 52.26 | 5.28 | 60.78 | 34 |
| Vicilin precursor, partial | CAA68708.1 | 1933.73 | 49.49 | 5.21 | 59.82 | 32 |
| Vicilin 47k | CBK38920.1 | 1443.46 | 49.92 | 5.28 | 43.76 | 27 |
| Vicilin, partial | CAF25232.1 | 1431.21 | 47.25 | 5.28 | 49.40 | 28 |
| Vicilin 47k | CBK38922.1 | 1390.83 | 49.69 | 5.47 | 52.51 | 29 |
| Ferritin | AAB24082.1 | 1367.35 | 23.62 | 5.50 | 50.00 | 13 |
| Vicilin 47k | CBK38917.1 | 1170.04 | 49.49 | 5.36 | 41.32 | 24 |
| Legumin A2 | P15838.1 | 1167.20 | 59.64 | 6.20 | 34.04 | 21 |
| LegA class precursor | CAA10722.1 | 1147.36 | 59.16 | 6.15 | 32.50 | 21 |
| Legumin | CAA26720.1 | 1063.18 | 57.99 | 6.09 | 33.33 | 21 |
| Ferritin | CAA51786.1 | 987.69 | 28.86 | 6.01 | 43.48 | 12 |
| Provicilin | P02854.1 | 976.04 | 46.41 | 5.26 | 38.54 | 20 |
| Provicilin, Type A | P02855.1 | 975.72 | 31.52 | 5.47 | 56.36 | 19 |
| Convicilin | CAB82855.1 | 787.07 | 72.14 | 5.38 | 25.45 | 17 |
| Convicilin, precursor | P13915.1 | 751.18 | 67.07 | 6.29 | 31.35 | 19 |
| Cvc, partial | CAP06315.1 | 627.98 | 62.16 | 5.58 | 26.00 | 17 |
| Legumin J | P05692.1 | 391.89 | 57.21 | 5.64 | 25.45 | 10 |
| Lectin | P02867.1 | 388.54 | 30.25 | 4.76 | 13.82 | 4 |
| Cvc, Partial | CAP06314.1 | 377.99 | 53.23 | 5.15 | 24.39 | 11 |
| Legumin K | P05693.1 | 369.99 | 39.83 | 5.28 | 29.43 | 8 |
| Convicilin, precursor | P13919.2 | 344.14 | 46.37 | 5.19 | 22.54 | 8 |
| Vicilin, 14 Kda component | P02856.1 | 284.10 | 14.03 | 5.13 | 41.94 | 6 |
| Albumin-2 | P08688.1 | 264.60 | 26.40 | 5.02 | 21.65 | 7 |
| Legumin (minor small) | CAA47809.1 | 214.01 | 65.18 | 5.30 | 10.60 | 4 |
| Alpha-Galactosidase 1 | CAF34023.1 | 177.29 | 45.40 | 6.59 | 12.84 | 4 |
| Acetyl-Coenzyme A Carboxylase Carboxyl Transferase subunit Alpha, Chloroplastic | Q41008.1 | 146.73 | 96.48 | 9.83 | 22.74 | 14 |
| Elongation Factor 1-Alpha | Q41011.1 | 136.15 | 49.55 | 9.60 | 18.57 | 7 |
| Seed Biotin-containing protein SBP65 | Q41060.1 | 124.36 | 59.63 | 5.88 | 23.59 | 11 |
| Gag/Pol polyprotein | AAQ82037.1 | 122.36 | 258.16 | 6.32 | 13.84 | 23 |
| Dehydrin 2 | AAB51380.1 | 116.52 | 27.13 | 6.56 | 28.24 | 5 |
| 110 kDa 4SNC-Tudor domain protein | BAC06184.1 | 107.27 | 108.61 | 7.67 | 18.10 | 17 |
| Acetyl-Coa Carboxylase, partial | BAA94752.1 | 103.50 | 91.37 | 9.69 | 20.36 | 12 |

[1]UniProt accession number;
[2]MASCOT protein score (index for determining accuracy identification by Mascot software);
[3]Calculated molecular weight. kDa;
[4]Calculated isoelectric point;
[5]Protein sequence coverage (%);
[6]Number of unique peptides matched to mass peaks.
