# Supplementary material for: Accumulation of storage proteins in plant seeds is mediated by amyloid formation
Source: PLoS Biol. 2020 Jul 23;18(7):e3000564. doi: 10.1371/journal.pbio.3000564 (PMC7377382; doi:10.1371/journal.pbio.3000564)
Supplement: S2 Table — Values for S2 Table are presented in the supporting information (S4 Data). (PDF) [file pbio.3000564.s010.pdf]

S2 Table. Content of the secondary structure elements (%*) in plant proteins and human Aβ-peptide (1-42 aa).

Protein state	Protein	α-helix	β-sheet	β-turn	Disordered
**Soluble proteins (50% HFIP)**	**Vicilin**	8±1	39±1	21±1	32±4*
	**Cupin-1.1**	52±3	4±1	18±3	26±3
	**Cupin-1.2**	38±1	12±1	20±2	30±3
	**Aβ**	53±2	7±1	16±2	24±4
**Fibrillar proteins (distilled water, 7 days after HFIP evaporation)**	**Vicilin**	4±1	41±3	20±2	35±6
	**Cupin-1.1**	4±1	40±1	21±2	35±4
	**Cupin-1.2**	3±2	42±2	23±2	32±1
	**Aβ**	3±1	40±1	20±1	37±2

*Results are shown as the mean (%) ± the standard deviation.
