# Supplementary material for: Accumulation of storage proteins in plant seeds is mediated by amyloid formation
Source: PLoS Biol. 2020 Jul 23;18(7):e3000564. doi: 10.1371/journal.pbio.3000564 (PMC7377382; doi:10.1371/journal.pbio.3000564)
Supplement: S3 Table — (PDF) [file pbio.3000564.s011.pdf]

S3 Table. Oligonucleotides used in this study.

Primer	Sequence 5'–3'
VicHindF	TACCAAGCTTATGAGATCCGATCAAGAGAACC
VicBamR	ATTAGGATCCCTCTCTTTGCAGAGGCTGA
CUP1(1)HindF	ATGTAAGCTTATGTATGAGAACGAAAACGGTCA
CUP1(1)BamR	TAACGGATCCTTCTATCTCCTCGTAATTGGT
CUP1(2)HindF	TACAAAGCTTATGAGAAGTCGCAATCCTATCTA
CUP1(2)BamR	TAACGGATCCATCAACCTCATGAGAAGATC
CDVicNot1F	TACTGCGGCCGCAAGATCCGATCAAGAGAACC
CDVicSal1R	ACGTGTCGACTTACTCTCTTTGCAGAGGCTGA
CDCUP1(1)Not1F	TACAGCGGCCGCATATGAGAACGAAAACGGTCA
CDCUP1(1)Sal1R	TAACGTCGACTTATTCTATCTCCTCGTAATTGGT
CDCUP1(2)Not1F	TACAGCGGCCGCAAGAAGTCGCAATCCTATCTA
CDCUP1(2)Sal1R	ACGTGTCGACTTAATCAACCTCATGAGAAGATC
AlicVicF	AGAAGGAGATATAACTATGAGATCCGATCAAGAGAACC
AlicVicR	GTGGTGGTGATGGTGATGGCCCTCTCTTTGCAGAGGCTGA
AlicCUP1(1)F	AGAAGGAGATATAACTATGTATGAGAACGAAAACGGTCA
AlicCUP1(1)R	GTGGTGGTGATGGTGATGGCCTTCTATCTCCTCGTAATTGGT
AlicCUP1(2)F	AGAAGGAGATATAACTATGAGAAGTCGCAATCCTATCTA
AlicCUP1(2)R	GTGGTGGTGATGGTGATGGCCATCAACCTCATGAGAAGATC
