# Supplementary material for: Accumulation of storage proteins in plant seeds is mediated by amyloid formation
Source: PLoS Biol. 2020 Jul 23;18(7):e3000564. doi: 10.1371/journal.pbio.3000564 (PMC7377382; doi:10.1371/journal.pbio.3000564)
Supplement: S1 Data — (PDF) [file pbio.3000564.s012.pdf]

S1 Data. Per-residue β-sheet propensity for the full-length Vicilin protein and its domains, Cupin-1.1 and Cupin-1.2.

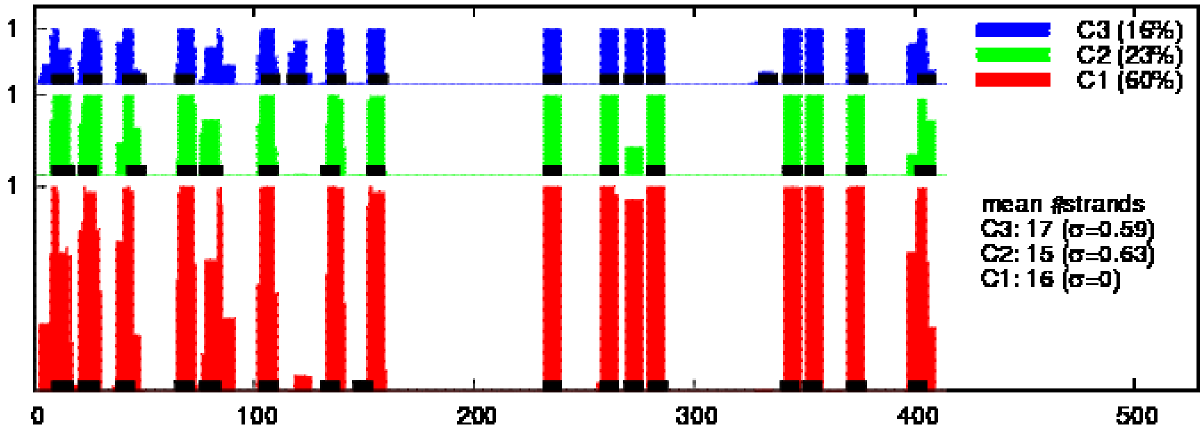

Fig 1. Per-residue β-sheet propensity for full-length Vicilin predicted by the AmyloidMutants[1] program. The x-axis indicates residue position. Each cluster ("C1","C2",...) is given its own color, one on top of another, with the relative size of that cluster in the ensemble indicated by its vertical size and the percentage in the legend. The largest cluster is the most probable structure. The y-axis the β-sheet propensity of each residue position, for each cluster. Black lines indicate the β-sheet assignment of a single representative mediod structure that is at the center of each cluster.

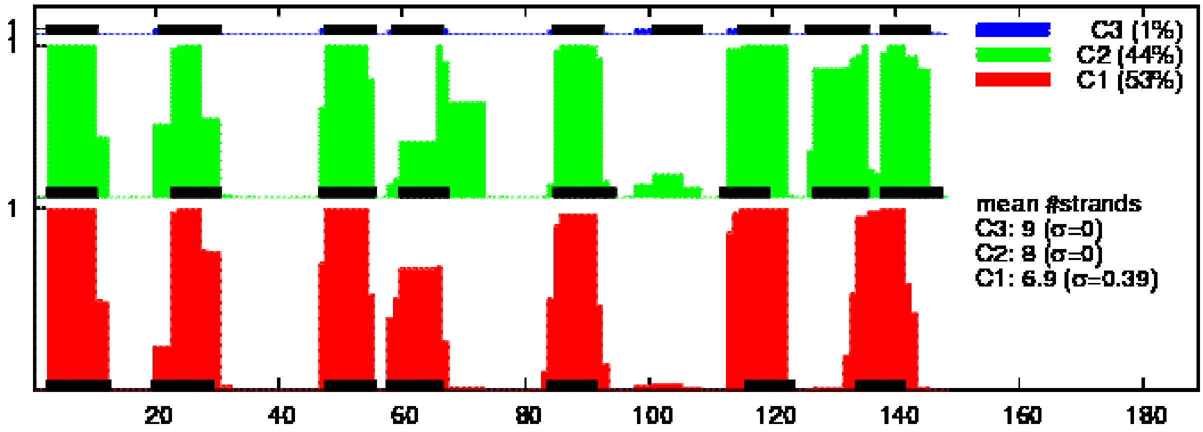

Fig 2. Per-residue β-sheet propensity for Cupin-1.1 predicted by the AmyloidMutants[1] program. The x-axis indicates residue position. Each cluster ("C1","C2",...) is given its own color, one on top of another, with the relative size of that cluster in the ensemble indicated by its vertical size and the percentage in the legend. The largest cluster is the most probable structure. The y-axis the β-sheet propensity of each residue position, for each cluster. Black lines indicate the β-sheet assignment of a single representative mediod structure that is at the center of each cluster.

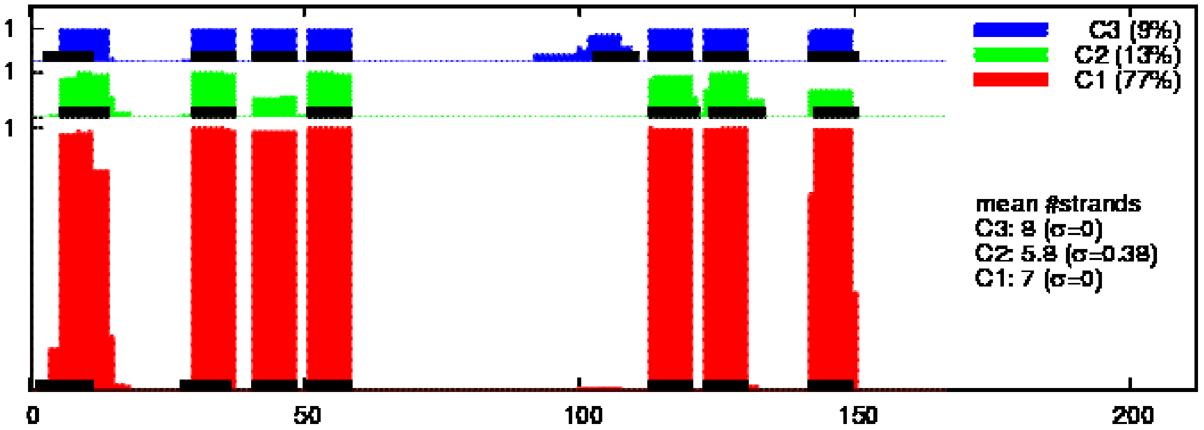

Fig 3. Per-residue β-sheet propensity for Cupin-1.2 predicted by the AmyloidMutants[1] program. The x-axis indicates residue position. Each cluster ("C1","C2",...) is given its own color, one on top of another, with the relative size of that cluster in the ensemble indicated by its vertical size and the percentage in the legend. The largest cluster is the most probable structure. The y-axis the β-sheet propensity of each residue position, for each cluster. Black lines indicate the β-sheet assignment of a single representative mediod structure that is at the center of each cluster.

Reference:

1. O'Donnell, C. W. *et al.* A method for probing the mutational landscape of amyloid structure. *Bioinformatics* (2011). doi:10.1093/bioinformatics/btr238
