# Supplementary material for: Accumulation of storage proteins in plant seeds is mediated by amyloid formation
Source: PLoS Biol. 2020 Jul 23;18(7):e3000564. doi: 10.1371/journal.pbio.3000564 (PMC7377382; doi:10.1371/journal.pbio.3000564)
Supplement: S2 Data — ThT, Thioflavin T. (PDF) [file pbio.3000564.s013.pdf]

Supplementary Data S2.pdf

Li - Ch1

256.0 x 256.0

Channel 2
MAX_Pea-12.12.18-2-0002.tif

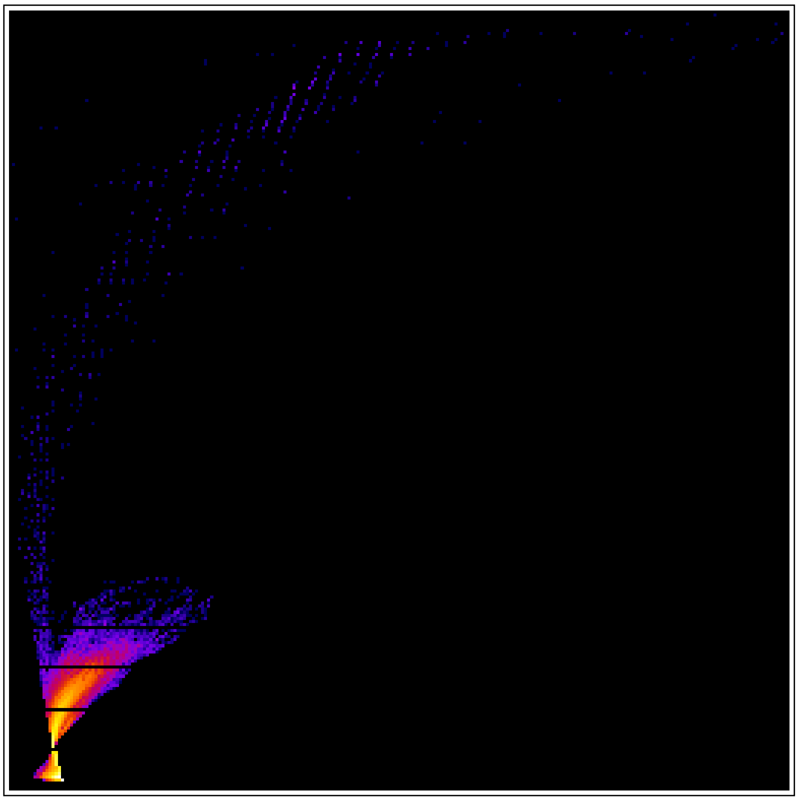

Channel 1
MAX_Pea-12.12.18-2-0001.tif

Li - Ch2

256.0 x 256.0

Channel 2
MAX_Pea-12.12.18-2-0002.tif

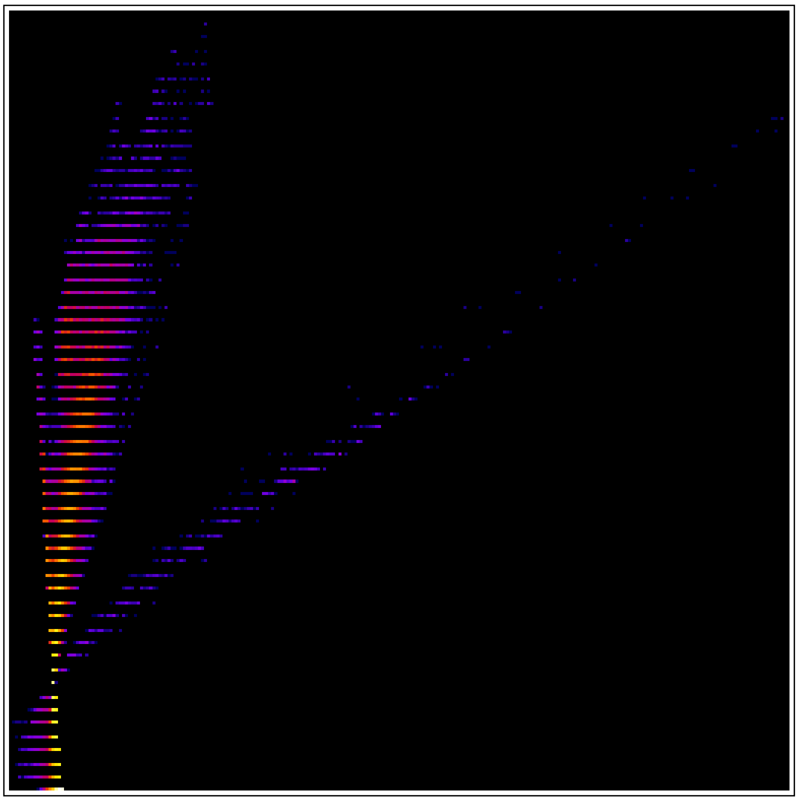

Channel 1
MAX_Pea-12.12.18-2-0001.tif

2D intensity histogram

256.0 x 256.0

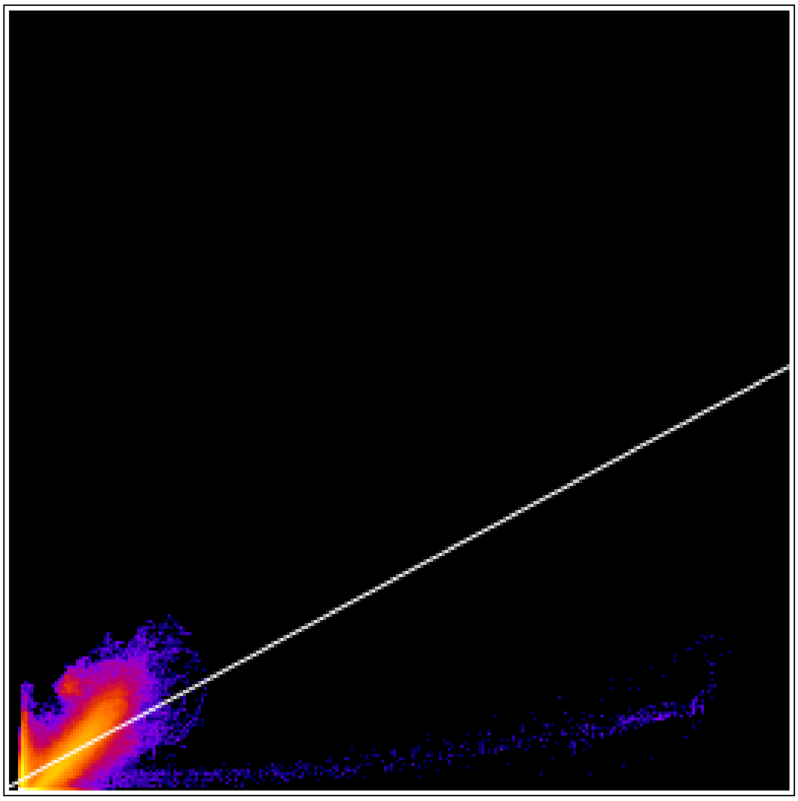

Channel 2
MAX_Pea-12.12.18-2-0002.tif

Channel 1
MAX_Pea-12.12.18-2-0001.tif

Smoothed & shuffled channel 1

1024.0 x 1024.0

Channel 2
MAX_Pea-12.12.18-2-0002.tif

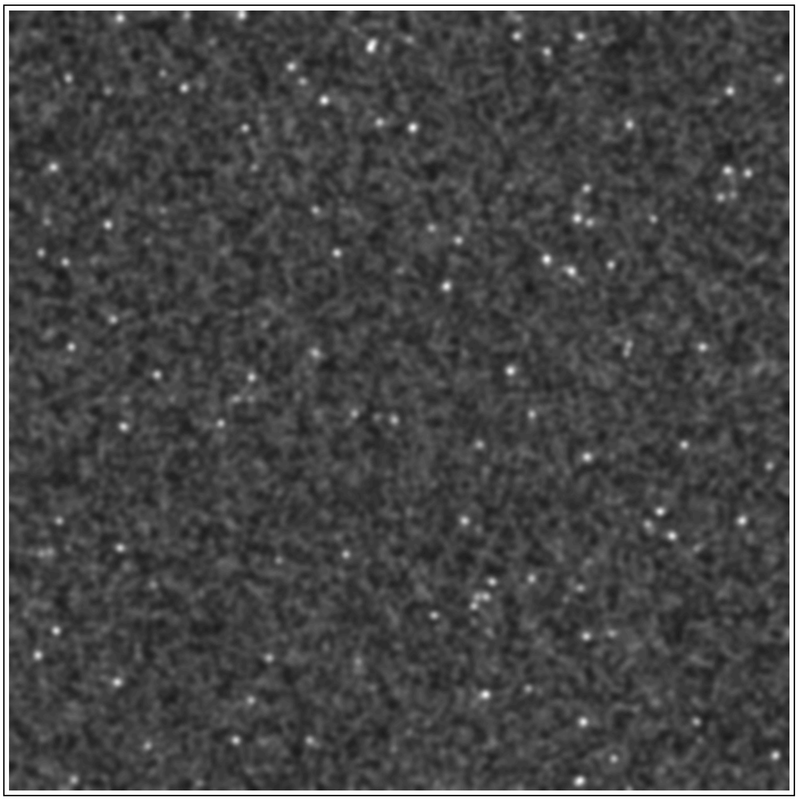

Channel 1
MAX_Pea-12.12.18-2-0001.tif

Channel 1 (Max Projection)

1024.0 x 1024.0

Channel 2
MAX_Pea-12.12.18-2-0002.tif

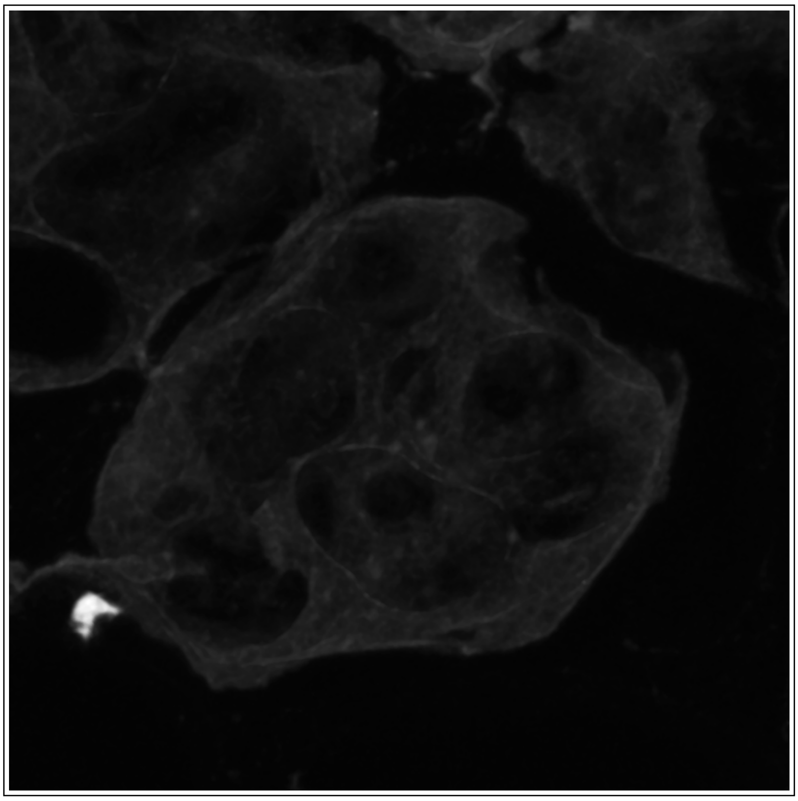

Channel 1
MAX_Pea-12.12.18-2-0001.tif

Channel 2 (Max Projection)

1024.0 x 1024.0

Channel 2
MAX_Pea-12.12.18-2-0002.tif

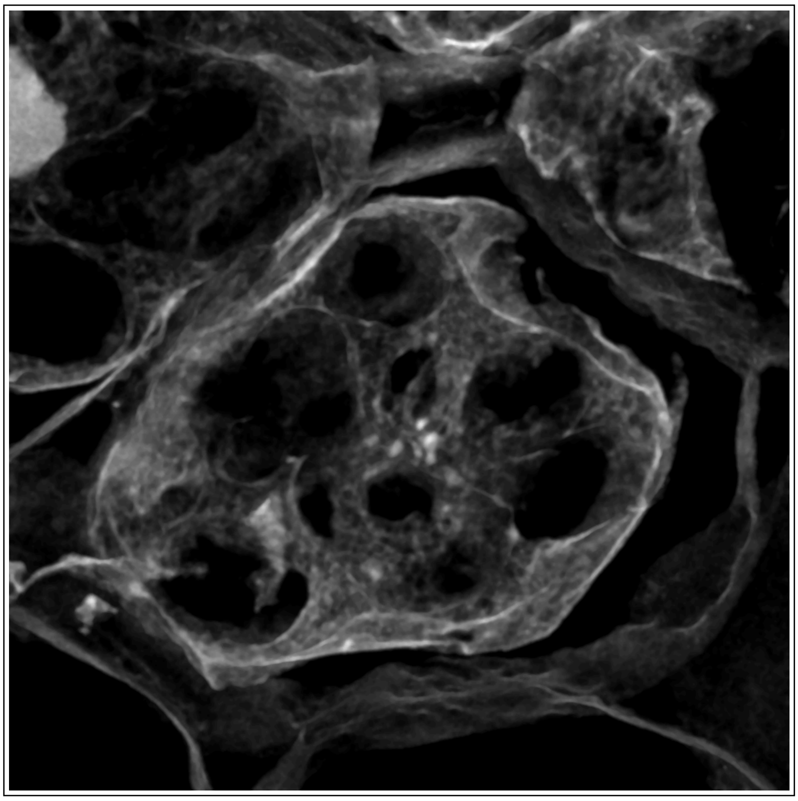

Channel 1
MAX_Pea-12.12.18-2-0001.tif

Coloc_Job_Name: Colocalization_of_MAX_Pea-12.12.18-2-0001.tif_versus_MAX_Pea-12.12.18-2-0002.tif_1396741106

% zero-zero pixels: 0.00

% saturated ch1 pixels: 0.00

% saturated ch2 pixels: 0.00

Channel 1 Max: 236.000

Channel 2 Max: 57.000

Channel 1 Min: 3.000

Channel 2 Min: 0.000

Channel 1 Mean: 13.512

Channel 2 Mean: 7.964

Channel 1 Integrated (Sum) Intensity: 14168173.000

Channel 2 Integrated (Sum) Intensity: 8351149.000

Mask Type Used: none

Mask ID Used: 1396741106

m (slope): 0.59

b (y-intercept): 0.04

b to y-mean ratio: 0.00

Ch1 Max Threshold: 12.00

Ch2 Max Threshold: 7.00

Threshold regression: Bisection

Pearson's R value (no threshold): 0.66

Pearson's R value (below threshold): 0.05
Pearson's R value (above threshold): 0.44
Li's ICQ value: 0.325
Spearman's rank correlation value: 0.73402428
Spearman's correlation t-statistic: 1106.7802
t-statistic degrees of freedom: 1048574.000
Manders' M1 (Above zero intensity of Ch2): 0.892
Manders' M2 (Above zero intensity of Ch1): 1.000
Manders' tM1 (Above autothreshold of Ch2): 0.709
Manders' tM2 (Above autothreshold of Ch1): 0.804
Kendall's Tau-b rank correlation value: 0.5936
Costes P-Value: 1.00
Costes Shuffled Mean: -0.00
Costes Shuffled Std.D.: 0.01
Ratio of rand. Pearsons >= actual Pearsons value : 0.00
