# Supplementary material for: Accumulation of storage proteins in plant seeds is mediated by amyloid formation
Source: PLoS Biol. 2020 Jul 23;18(7):e3000564. doi: 10.1371/journal.pbio.3000564 (PMC7377382; doi:10.1371/journal.pbio.3000564)
Supplement: Attachment — Submitted filename: Response_to_reviewers.docx [file pbio.3000564.s017.docx]

**RESPONSE TO THE COMMENTS OF THE REVIEWERS:**

**REVIEWER #1:

This manuscript reports that seeds of the garden pea Pisum sativum contain amyloid-like aggregates and that the most abundant storage protein, Vicilin, is able to assemble into amyloid structures. The data support the presence of amyloids in vivo and in vitro. The stability of the amyloid structures changes with seed maturation and assemblies disappear at seed germination.

The authors have identified Vicilin as being resistant to treatment with SDS during isolation from pea seeds. Tryptic digestion followed by mass spectrometry indicates that the full-length protein is present in detergent-insoluble polymers.
Experiments with full-length (FL) or the two isolated cuprin domains indicate that these proteins are able to form fibrils with amyloid character, when incubated in vitro under denaturing conditions. Some fibrillar material is observed under relatively mild conditions, while treatment with HFIP, known to induce secondary structure, leads to the formation if extensive fibrillar structures.

Secretion of the proteins on the surface of the E. curli cells was suggestive of the formation of amyloid-structures aggregates by these proteins. The proteins were also expressed in S. cerevisiae and demonstrates intracellular aggregation. However, many aggregation-prone proteins may form puncta in vivo in yeast. A key reported finding is that vicilin and ThT-positive structures are found co-localised in mature seeds in vivo.

The authors suggest that this is the first demonstration of functional amyloids in plants. This would certainly be a novel and significant finding, since no functional amyloids have been reported in plants to date – although several plant proteins have been shown to form amyloids under denaturing or modifying conditions.

Some work has even suggested that plants may actively produce small molecule inhibitors of fibrillation – although this study suggests that the Vicilin amyloids are associated with storage within seeds and that they disappear upon germination.

However, the work is not described in sufficient detail, nor are the different protein preparations characterised sufficiently well, to fully support the findings as they currently are presented in the manuscript. The experimental design is appropriate and the authors have employed a wide variety of approaches to validate the findings. However, more detail about experimental methods is required to provide confidence in the results.

Points to be addressed:**

**P2      2nd sentence in 2nd paragraph. Change to” Amyloid deposition is associated with the development… because in many conditions, the direct link between amyloid deposition and pathogenesis is unclear.**
- We thank Reviewer for their comment. This sentence has been changed (Page 3, line 70).

**Include further mention of many plant proteins that have been shown to be capable of forming amyloid structures under denaturing conditions:**
[**https://www.sciencedirect.com/science/article/abs/pii/S0301462218300139**](https://www.sciencedirect.com/science/article/abs/pii/S0301462218300139)
[**https://www.sciencedirect.com/science/article/pii/S0014579399007899**](https://www.sciencedirect.com/science/article/pii/S0014579399007899)

- Thank you for this comment, we have added this information and additional references to the introduction section (Page 3, lines 87-90).

**The authors should also discuss the early suggestions that plants were rich in amyloids – although it seems likely that these were carbohydrate-rich structures that were characterised in this way, since amyloid-specific stains were not used. See KOOIMAN, P. Amyloids of Plant Seeds. Nature 179, 107–109 (1957) and other associated publications.**

- Thank you for this comment. Initially, this term had been attributed to cell wall carbohydrates further identified as xyloglucans. We have added this story to Introduction due to its historical value (Page 3, lines 87-90).

**No detail is provided regarding the protocol used for refolding proteins from the denaturing conditions used for protein purification. What methods were used to determine that proteins had refolded correctly?** **The reference to the Methods in Enzymology protocol is for purification only and does not include not sufficient to verify that proteins were produced correctly and refolded to a native, monomeric state.**
- First of all, it should be noted that the questions of which state of Vicilin is native and what processes precede its fibrillation are not fully understood. A series of studies of the seed storage proteins in various plant species has demonstrated that most of them including 7S Globulins, to which Vicilin belongs, can form trimers in vivo (Czubinski, J. *et al*., 2015, Acta Crystallogr. D Biol. Crystallogr. DOI: 10.1107/S1399004714025073). Overall, it is unclear whether monomeric, oligomeric or polymeric state (or all of these states) is native for Vicilin. Typically, refolding is used when protein has functional activity (for example, enzymatic) that can be monitored after refolding to verify its correctness. It is impossible for Vicilin. By this reason, we did not use refolding protocols in this work. Also, for the formation of amyloid fibrils under physiological conditions *in vivo* additional cofactors (for example, chaperons) may be required that are absent *in vitro*. Overall, the goal of the experiments *in vitro* is to check whether proteins (Vicilin and its domains) are capable of forming fibrils.

In our work, purified proteins were dissolved in a mixture of an organic HFIP solvent and water with HFIP being subsequently removed from the sample. This solvent (often,100 % HFIP) is widely used to ensure that proteins and peptides are entirely monomeric for further analogical aggregation experiments. Moreover, a method was previously developed for the standardized and biocompatible preparation of aggregate-free amyloid beta peptide for biophysical and biological studies of Alzheimer's disease (Broersen, K. *et al*., 2011, A standardized and biocompatible preparation of aggregate-free amyloid beta peptide for biophysical and biological studies of Alzheimer's disease, Protein engineering design & selection), one of the stages of which is the dissolution of the peptide in HFIP. Considering the fact that Vicilin, Cupin-1.1 and Cupin-1.2 are poorly soluble in common buffers, we suggest that the use of HFIP to dissolve protein with its subsequent evaporation (that is, fibrillogenesis occurs in distilled water, not in HFIP) minimally affects structure and properties of the protein. Additional information on experimental procedures has been included to Materials and Methods section (Pages 27-28, lines 791-845) and clarified in Results (Pages 5-8).

**What is meant by “Proteins were concentrated by ethanol? Details should be provided. Methanol is used in the MinE reference.**

- The same protocol as in the Serio et al., Meth. Enz., 1999 paper has been used with one exception: methanol has been substituted for ethanol. This issue has been included in Materials and methods (page 27, lines 788-789).

**What effect does 50% HFIP have on the CD spectra?**
- As we have already noted, the use of 50% HFIP is a widely accepted approach for dissolving proteins for further aggregation experiments. Our experimental data for other objects, such as RopA and RopB amyloid proteins of the root nodule bacterium *Rhizobium leguminosarum* (Kosolapova *et al*., 2019, Two Novel Amyloid Proteins, RopA and RopB, from the Root Nodule Bacterium Rhizobium leguminosarum. Biomolecules 2019, 9, 694), indicate that protein molecules have partially ordered structure after dissolving in 50% HFIP buffer. Here we compared far-UV circular dichroism (CD) spectra of Vicilin, Cupin-1.1, Cupin-1.2 dissolved in 50% HFIP (Figure 2a, left) and in distilled water where these proteins were incubated for 7 days after HFIP evaporation to obtain fibrils (Figure 2a, right). A significant change in the CD spectra of the samples during fibrillogenesis has been detected. Samples of the Cupin-1.1, Cupin-1.2 and Vicilin fibrils obtained in distilled water have demonstrated apparent negative shoulder around 220-230 nm (Figure 2a, right) that allows us to suggest the essential content of β-sheets in their structure. This spectral minimum has been less pronounced for Vicilin aggregates that does not contradict to the results of TEM indicating a lower content of highly-ordered fibrillar structures in the Vicilin sample compared to the Cupin-1.1 and Cupin-1.2 samples (Figure 1).

It has also been shown that the Cupin-1.1 and Cupin-1.2 proteins dissolved in 50% HFIP have exhibited relatively low β-sheet content (4-12%) in comparison with Vicilin (39%) (Table S2). Further formation of the Vicilin, Cupin-1.1 and Cupin-1.2 fibrils in water after HFIP evaporation have resulted in the increase of the β-sheet content (40-42%) in their structures that was approximately the same as for the human Aβ-peptide (1-42 aa) fibrils (40%) used as the positive control (Table S2).

**Why does the Cupin-1.2 run so large on the gel in both boiled and unboiled samples? (fig 1c)**

- Probably, this effect could be caused by high protein concentration. We have obtained novel western blots with freshly prepared proteins and the size of Cupin-1.2 was as usual. We have corrected this image (Fig 1c).
**Replace “fibrillary” with “fibrillar” throughout. This is a Microsoft Word auto-correct error.**
- We thank the Reviewer for their comment. These typos have been corrected.

**Absorption not Absorbtion in Figure 2d.**
- We thank the Reviewer for their comment. It has been corrected.

**While there is some overlap of the signals from ThT and Vicilin (Fig. 5a-c), there are many structures that are strongly ThT-positive but do not show Vicilin co-localisation. What are these structures? Some analysis of the degree of co-localisation should be performed, e.g. with Coloc2 in ImageJ.**
- We performed colocalization analysis that demonstrated strong co-localization between Vicilin and ThT signal in the cytoplasm (Supplementary data S2-S3). Strong ThT fluorescence of the plant cell walls without anti-Vicilin signal suggests the presence of amyloid aggregates of other proteins in their structure as it has been described for fungal cell walls (Kalebina *et al*., Prion, 2008; Ryzhova *et al*., Curr. Genet, 2018) and represent interesting subject for further studies.

**The image in Fig 5d-f is difficult to evaluate. A bright field image should be included. The staining of these structures cannot be evaluated at this magnification.**

- We have included brigthfield image as well as detailed analysis of the presence of amyloid fibrils in protein bodies by TEM with immunogold (Figure 5i-n and lines 461-484).

**TEM analysis of these isolated, purified protein bodies should be performed and included, since the mild purification should allow retention of fibrillar structures.**

- Thank you, we have performed such analysis and have detected fibrillar structures that bind anti-Vicilin antibody after membrane removal from protein bodies (Figure 5i-m, lines 469-480).

**SDS PAGE analysis of these purified bodies should be conducted, with silver staining and/or Coomassie to indicate relative content of Vicilin, and other proteins, in these preparations.**

- We have included images of Coomassie staining gel with proteins extracted from protein bodies as well as western blot image (Figure 5n, lines 480-484).

**Is it surprising that antibodies raised against purified Vicilin react strongly with the amyloid form? They also bind the fragments after proteolytic digestion. It is not clear in the Methods section how the protein was prepared for immunisation. Which protocol was used? How was it verified that the antibodies were specific for Vicilin? If recombinant protein was used, then was it raised against full-length protein?**

- The Recombinant, full-length Vicilin was used for rabbit immunization. This polyclonal antibody recognizes full-length Vicilin and both its domains and does not exhibit cross-reactivity against E. coli proteins (Figure S8). Being solubilized Vicilin immediately aggregates in PBS (Figure 1). Thus, Vicilin solution used for immunization contained not only monomers of Vicilin but also its aggregates that explains the ability of this polyclonal antibody to recognize both monomers and polymers of Vicilin.

**Further discussion should be included regarding the differences in proteolytic resistance of the material from total protein lysates and fibrils formed from purified recombinant proteins.**

- We have included several sentences in the discussion regarding these differences (pages 23-24, lines 661-667).

**What is the likely cause of the protolytic degradation observed from 20 days onwards, i.e. loss of M band?**

- Probably, loss of M band could be caused not only by proteolytic degradation but by amyloid formation that leads to incorporation of the full-length Vicilin monomers into amyloids that are poorly dissolved by SDS while proteolytic products are remaining soluble.

**Further discussion should be included about the likely differences in protease-resistant aggregates between pea seeds and commercial canned products. For example, what processing occurs that could account for the differences?**

- We have included several sentences in the Discussion (page 24, lines 667-669). We think that these differences are explained by pre-treatment of canned products at high temperatures that partially dissolve Vicilin aggregates thus allowing their more efficient entrance in the gel.
-------

**REVIEWER #2:**

**Manuscript by Antonets et al. investigates the amyloid properties of plant proteins. It was previously predicted that the Vicillin protein from the seeds of the garden pea Pisum sativum L shows amyloid like properties. In this study  authors go on to show that Vicillin protein that comprises of 2 subunits, Cupin 1.1 and Cupin 1.2 , show amyloid like properties. Authors determine that Cupin 1.2 shows more fibrillar nature  while Cupin1.1 and full vicillin protein show aggreagates. Authors conclude using Circular Dichroism, ThioflavinT,microscopy to prove that Vicillin amyloid properties. Although the study is interesting, some of the results and their interpretations do not fully justify the overall conclusions suggested by the authors. More detailed analysis and mechanistic studies showing the residues important for how Vicillin establishes fibrils would strengthen the manuscript.  One of the more interesting findings is the cytotoxic nature of the fibrils. These results
would benefit from the mechanistic mutations showing abrogation of fibrils lead to abrogation of the cytotoxic nature of Vicillin.**

**Major comments

1- Once amyloid proteins are polymerized they are resistant to degradation. Even though they are boiled in SDS, they don’t depolymerize into monomers and therefore do not enter a 12% SDS-PAGE gel. The fact that the authors show that the Vicillin enters the gel is counterproductive to their argument that this is an amyloid protein.  Can the authors comment on this?**

- The ‘polymers’ fraction in all images indicates protein aggregates remained in the wells after electrophoresis or only slightly migrated to the stacking (not resolving) gel indicating that boiling in SDS reduces their size. We venture to disagree with Reviewer that all amyloids are resistant to hot SDS treatment. To our knowledge, most of known amyloids including majority of human amyloids and yeast amyloid prions are resistant to cold SDS but solubilize in hot SDS (see, for example, Kushnirov *et al*., Purification and analysis of prion and amyloid aggregates. Methods. 2006;39: 50–55. pmid:16774835; Nizhnikov *et al*., PLOS One, 2014, Table 6). Other amyloids like human huntingtin exon-1 expanded (Hazeki *et al.,* Biochem. Biophys. Res. Comm., 2000) and CsgA curlin (Chapman *et al*., Science, 2002) are resistant to treatment with hot SDS but solubilize in formic acid. Vicilin aggregates are partially soluble in hot SDS and dissolve in formic acid (Fig S6) that agrees with their amyloid nature as well as results of their staining with amyloid-specific dyes, TEM, protease resistance and other experiments including incorporated after revision X-ray diffraction study.

**2- Figure 1, the TEM pictures only show fibrillar structures in Cupin 1.2. Have the authors tested other buffers here such as potassium phosphate buffer that supports the formation of amyloid fibrils in vitro?**
- We have tested different conditions for amyloid fibrils formation *in vitro*. In particular, we have used 5 mM potassium phosphate buffer (Fig. 1 b, row 1) with prolonged incubation (Fig. 1 b, row 2), high temperature (Fig. 1 b, row 3) and 3 M denaturing agent guanidine hydrochloride (data not shown), but all these conditions allowed us only to show fibrillary structures for Cupin-1.2. We suggested that this is due to the fact that Vicilin and Cupin-1.1 are not well dissolved under conditions selected. We have used 50% organic HFIP solvent to solubilize these proteins. After removing HFIP from the samples and incubation in distilled water, Cupin-1.1, like Cupin-1.2, predominantly formed fibrillar structures (Fig. 1 b, row 4). The Vicilin sample was a mixture of fibrillar and non-fibrillar aggregates. In order to shift the equilibrium towards the formation of the Vicilin fibrils, we used the ‘seed’ obtained on the basis of the previously prepared Vicilin mixture of fibrillar and non-fibrillar aggregates, Cupin-1.1 fibrils or Cupin-1.2 fibrils. Using the Vicilin and Cupin-1.2 ‘seeds’ allowed us to obtain *bona fide* the Vicilin amyloid fibrils (Fig. 3 a, left and right Panels). The descriptions of conditions for producing amyloid fibrils have been more clearly described in Figures 1 and 2.

**3- HFIP is used to break the amyloid fibrils into its monomers. The fact that HFIP usage leads to higher ThT levels is puzzling. Are there any other examples in amyloids that show a similar characteristic? It would be good to use a positive control not only for this assay but other assays as well.**
- Indeed, HFIP is used to dissolve proteins in a sample. Moreover, a method has been previously developed for the standardized and biocompatible preparation of aggregate-free amyloid-beta peptide for biophysical and biological studies of Alzheimer's disease (Broersen et al., 2011. A standardized and biocompatible preparation of aggregate-free amyloid beta peptide for biophysical and biological studies of Alzheimer's disease. *Protein engineering design & selection*, *24*(9), 743-750. https://doi.org/10.1093/protein/gzr020), one of the stages of which is the dissolution of the peptide in HFIP. In our work, we also used 50% HFIP to dissolve target proteins to monomers. We showed that values of ThT fluorescence in HFIP in the free state and in the presence of monomeric proteins are similar (Fig. 1g). The formation of amyloid fibrils from dissolved proteins occurred in distilled water after evaporation of HFIP from the sample and it was accompanied by a significant increase in ThT fluorescence (Fig. 1h). Similar technique has already been used previously in other works to obtain amyloid fibrils, in particular, from the human amyloid-beta peptide and RopA and RopB proteins from the root nodule bacterium *Rhizobium* leguminosarum (Kayed, R. *et al*., 2007, Fibril specific, conformation dependent antibodies recognize a generic epitope common to amyloid fibrils and fibrillar oligomers that is absent in prefibrillar oligomers. Mol Neurodegeneration 2, 18, <https://doi.org/10.1186/1750-1326-2-18>; Kuznetsova, I.M. *et al.*, 2012, A New Trend in the Experimental Methodology for the Analysis of the Thioflavin T Binding to Amyloid Fibrils. Mol Neurobiol 45, 488–498, https://doi.org/10.1007/s12035-012-8272-y; Kosolapova, A.O. *et al.*, 2019, Two Novel Amyloid Proteins, RopA and RopB, from the Root Nodule Bacterium *Rhizobium leguminosarum*, Biomolecules, 9, 694). We have added experiments with human Aβ-peptide (1-42 aa) as the positive control. We have corrected the designation of the experimental conditions on the Figures and their description in the text from "HFIP" to "after HFIP evaporation" to avoid misinterpretation by readers. Also, information on HFIP evaporation has been more clearly described in Material and Methods.

**4- Figure 2. ThT polymerization curves using recombinant proteins would help the readers to understand the polymerization kinetics of these molecules.  Also calculation of lag times could help identify the differences in Vicillin and its subunits. In its current form the ThT experiments are not very informative**
- First of all, we would like to note that the remark on the lack of information on the static ThT data is not fair. A shift in the absorption and fluorescence excitation spectra of ThT as well as a substantial increase in the fluorescence quantum yield and lifetime of the dye upon binding to the studied aggregates confirm their amyloid nature. The presence of two peaks in the fluorescence excitation spectra of ThT bound to aggregates indicates their heterogeneity (the presence of two types of aggregates in the samples interacting differently with ThT). Finally, close values of the fluorescence quantum yields and lifetimes of ThT bound to the Vicilin, Cupin-1.1, and Cupin-1.2 fibrils indicate a similarity of the dye binding sites, and therefore, a similar structure of amyloid fibers formed from Vicilin and its fragments. We have included additions on this subject in the text of the manuscript.

The study of the kinetics of amyloid fibrils formation under chosen conditions is not a trivial task, since the proteins are initially incubated in 50% HFIP, with further HFIP removal from the sample by evaporation in a stream of nitrogen. In this case, a simultaneous decrease in the HFIP content in the sample and increase in proteins concentration occur. Since this process takes 30 minutes or more (depending on the volume of the sample), significant changes in the sample can occur during the evaporation of HFIP. Despite our concerns, in accordance with the Reviewer's recommendation, we have conducted Vicilin, Cupin-1.1 and Cupin-1.2 polymerization study using ThT (Fig. 1e) after HFIP evaporation. In addition, we have performed similar experiments with Aβ-peptide (1-42) for comparison. We have shown that fluorescence of ThT in the presence of monomeric proteins before HFIP evaporation practically does not differ from free dye fluorescence (Fig.1g). This observation has proven a lack of the dye interaction with monomeric proteins. At the same time, ThT fluorescence significantly increases immediately after HFIP evaporation (Fig. 1h). The obtained results confirmed that, unfortunately, under the conditions chosen, the initial stages of fibrillogenesis cannot be reliably detected and lag times cannot be determined. This assumption has been proven by the TEM images of probes after HFIP evaporation (Fig. 1f). It should be noted that ThT kinetic data under these conditions for the plant proteins and well-studied human amyloid-beta peptide turned out to be similar. This discussion has been added to the text of the article.

**5-Manuscript benefit from the comparison of Cupin 1.1 and Cupin 1.2 amino acid sequence comparisons and the mechanistic explanation for why Cupin 1.2 forms fibrils instead of aggregates like vicillin and cupin 1.1. Would the alanine substitution of the predicted residues important for beta sheet fibrillar structure  change the amyloid readouts and the structure of these proteins?**

- Thank you, we have incorporated more detailed comparison of the Cupin-1.1 and Cupin-1.2 amino acid sequences in the text. These domains share very little at the level of amino acid sequence exhibiting only 21.8% identity and 33.0% similarity. Moreover, they significantly differ in their physicochemical properties: isoelectric points (pI) of Cupin-1.1 and Cupin-1.2 are 5.92 and 4.32, correspondingly (pI of the full-length Vicilin is 5.18). Nevertheless, according to the predicted with I-TASSER server, both Cupin-1.1 and Cupin-1.2 as well as full-length Vicilin form β-sheet-rich barrels (Figure 1a). Interestingly, all potentially amyloidogenic regions predicted by using AmylPred2 method in the Vicilin protein were located within boundaries of the Cupin-1.1 (regions indicated as R1-R5) and Cupin-1.2 (R6-R9) domains (Figure 1a). Moreover, R6-R9 regions of Cupin-1.2 were tightly stacked in β-sheets (Figure 1a) that may explain its higher propensity to form fibrils.

Futhermore, AmyloidMutants program has predicted the ability of Vicilin and both Cupins to form juxtaposed β-strands assembling into β-sheets (Data S1). The probability of such β-sheet formation was highest for Cupin-1.2 (Data S1). According to the AmyloidMutants, the Vicilin protein with all amyloidogenic regions substituted for alanine, would retain the ability to form amyloid fibrils though probability of such pleated β-structures formation in the alanine-substituted Vicilin decreases from 60 to 41% in comparison with the wild type Vicilin.

**6- Amyloid proteins show around 90% beta sheet conformation. Can the authors comment if Vicillin is showing only 40-50% beta sheets by CD, would it be described as a true amyloid? This can be included in the discussion.**
- We disagree with the statement that all amyloid fibrils are characterized by 90% beta sheet conformation. The content of beta sheets in *bona fide* fibrils formed from other proteins is often the same or even lower than shown in our work (see, for example, Ghosh *et al*. Structure based aggregation studies reveal the presence of helix-rich intermediate during α-Synuclein aggregation. Sci Rep 5, 9228 (2015). <https://doi.org/10.1038/srep09228>; Takunori Harada, Reiko Kuroda. CD measurements of β‐amyloid (1–40) and (1–42) in the condensed phase. 2010. Biopolymers, 95(2), 127–134. doi:10.1002/bip.21543; Juárez *et al*. Existence of Different Structural Intermediates on the Fibrillation Pathway of Human Serum Albumin. 2009. Biophys J Volume 96, Issue 6, Pages 2353-2370). The content of beta-sheets in amyloid-forming proteins depends on the structural features of the native protein, its size, the length of the amyloidogenic fragment, the preliminary effect on the native protein structure, etc. Moreover, we believe that the content of 90 % beta-sheets in fibrils is an exceptional case and is possible only for very short amyloid-forming peptides. Considering the fact that target proteins, and especially Vicilin, are large proteins, the detected content of beta-sheets in their fibrillar form does not cause contradictions. For amyloid fibrils formed from Aβ-peptide (1-42) we obtained the same results (about 40% beta sheets). These results have been added to Figure 2a and Table 2S with the discussion in the manuscript.

At the same time, we agree that when evaluating the content of the secondary structure in amyloid fibrils, there may be pitfalls. In particular, in the studied samples (as in the case of other amyloid fibrils), significant light scattering was observed, which, according to existing concepts, can distort the shape of the recorded CD spectra. Moreover, the standard basic sets of proteins used to estimate the secondary structure content are not representative for the analysis of the spectra of protein aggregates. In this regard, numerical estimates of the number of beta sheets in the sample should be treated with caution. For this reason, we additionally conducted a visual analysis of the recorded spectra using CD spectra of proteins and peptides with representative secondary structures. This analysis shows apparent negative shoulder around 220-230 nm (Figure 2a), that allows us to suggest the essential content of beta-sheets in their structure being characteristic of a true amyloid. We have added discussion of the results obtained by CD spectroscopy to the manuscript.

**7- As the amyloids adopt a fibrillar structure their toxicity decreases (e.g. beta-amyloid). The fact that the vicillin fibrils show toxicity is biologically interesting. Would disrupting the fibrillar structure mechanistically disrupt the cytotoxic nature?**
- Thank you, we have performed the experiment using the disrupting Vicilin fibrils by sonication that resulted in the significant loss of toxicity (Figure S7, lines 552-555). Thus, indeed, toxicity of Vicilin and both its domains is associated with their amyloid states.

**Minor Comments:**

**1.      Abstract would benefit from the description of findings on Cupin 1.1. and 1.2.**

- Thank you, we have added information on Cupin-1.1 and 1.2 to the abstract (lines 45-47).

**2.      Supplementary figures 3-6 lack the information whether these images are western blots and which antibodies are used.**
- Thank you for this comment, this information has been added.
-------

**REVIEWER #3:

The manuscript by Antones et al., presents exciting results, showing that similarly to Eukarya bacteria and archea, plant seeds also produce proteins that potentially harbor an amyloid fold. The authors use various commonly acceptable methods to prove this point, including resistance to SDS treatment, ThT and Congo Red binding, cross-seeding, and toxicity for yeast and mammalian cells. Building on their previous bioinformatics work, they have performed an extensive in vitro characterization of the protein Vicilin and they have further showed that ThT binding co-localizes with Vicilin in vivo and that certain forms of Vicilin are toxic for yeast and mammalian cells.

Major comments:

While the authors use a variety of methods to show that plant seeds proteins exhibit an amyloid fold, these methods are rather indirect and not quantitative and they lack some more structural insight that could have been obtained by more quantitative methods such as NMR, and (electron or x-ray) diffraction methods. The data presented in the paper leans heavily on such indirect evidence for amyloid formation, but being the pioneers to come up with the claim that plant seeds produce amyloid proteins, I would expect the (nicely studied) indirect results to be accompanied with stronger structural evidence of amyloid formation.**

- Thank you for this comment, we have performed X-ray diffraction analysis of Vicilin and its domains that has revealed circular diffuse X-ray reflections 4.7 Å and 10 Å for the Vicilin fibrils (Figure 3f), circular X-ray reflections 4.7-4.88 Å and 8.6 Å for the Cupin-1.1 fibrils, 4.7-4.9 Å and 8.8 Å for the Cupin-1.2 fibrils (Figure 3f). These reflections are typical for *bona fide* amyloid fibrils with the cross-β sheet quaternary structure. The 4.7-4.9 Å reflection is assumed to arise from the periodicity of the hydrogen-bonded β-strands oriented near perpendicular to the fiber axis, and the diffraction in the region of ∼8-11 Å is presumed to relate to the stacking of these sheets parallel to the fiber axis.

**Additional questions and comments:**

**1.  The CD results (figure 2) are not conclusive. It is very difficult to determine from this data that the proteins exhibit a beta sheet structure, let alone a cross beta sheet structure. Some CD curves (Vicilin) would probably fit better a random coil rather than a beta sheet structure, as is also indicated in the text. To the very least, I suggest to use a few secondary structure analysis software (such as Dichroweb, Biotools) in order to deconvolute the CD spectra and extract the contribution of different secondary structures to each curve. Some percentage of 'ordered beta sheet' is referred to in the text, but it is not clear what is the origin of this number**

- We agree with the Reviewer that in CD spectrum of Vicilin aggregates presented in the article the shoulder characteristic for beta-sheets is significantly smaller than the shoulder that is typical for unfolded proteins. This can be caused by the presence of non-fibrillar aggregates along with fibrils in the sample. We tried to increase the amount of fibrillar structures by changing the protocol for HFIP evaporation (changing the evaporation rate, residual sample volume) and protein concentration. This allowed us to obtain a larger fraction of fibrillar aggregates in the sample, and, therefore, the CD spectrum more characteristic for structured proteins (Fig. 2a). For estimation of protein secondary structure from CD spectra CDPro software, containing three popular methods (CONTIN, SELCON3, and CDSSTR) (Sreerama, N.; Woody, R. W. Estimation of protein secondary structure from circular dichroism spectra: comparison of CONTIN, SELCON, and CDSSTR methods with an expanded reference set. Anal Biochem 2000, 287, 252–260) and several basic sets of proteins with a known secondary structure (that include from 37 to 56 soluble, membrane, and denatured proteins with different content of the secondary structure), were used. The results obtained by different methods are summarized and averaged in Table S2. Despite the differences in the secondary structure of proteins upon dissolution in HFIP, after its evaporation the content of beta-sheets (40-42%) and beta-turns (20-23%) turned out to be quite high for all samples, which confirms the amyloid nature of aggregates formed from the target proteins. Similar results were obtained for amyloid fibrils formed from Aβ-peptide (1-42) (Table S2).

**2.      The ThT data is static, but a kinetic study of aggregation is missing as it will allow the reader to compare the aggregation kinetics of the plant proteins with ThT kinetic data obtained with well-studied common amyloid proteins (a beta, alpha synuclein, curli).**

- The study of the kinetics of amyloid fibrils formation under chosen conditions is not a trivial task, since the proteins are initially incubated in 50% HFIP for the proteins dissolution, then HFIP is removed from the sample by evaporation in a stream of nitrogen. In this case, a simultaneous decrease in the HFIP content in the sample and increase in proteins concentration occur. Since this process takes 30 minutes or more (depending on the volume of the sample), significant changes in the sample can occur during the evaporation of HFIP. Despite our concerns, in accordance with the Reviewer's recommendation, we conducted Vicilin, Cupin-1.1 and Cupin-1.2 polymerization study using ThT (Fig. 1) after HFIP evaporation. In addition, we have performed similar experiments with Aβ-peptide (1-42). We showed that fluorescence of ThT in the presence of monomeric proteins before HFIP evaporation practically does not differ from free dye fluorescence (Fig. 1g). At the same time ThT fluorescence increases significantly immediately after HFIP evaporation (Fig 1h). The obtained results confirmed that, unfortunately, under the chosen conditions the initial stages of fibrillogenesis cannot be reliably detected. This assumption has been proven by the TEM images of probes after HFIP evaporation (Fig. 1f). It should be noted that ThT kinetic data in these conditions for the plant proteins and well-studied common amyloid turned out to be similar. This discussion has been added to the text of the article.

**3.      It is not clear from the text what was the purpose of the fluorescence decay experiments.**

- Fluorescence decay experiments have been carried out to determine the fluorescence lifetime of ThT bound to studied aggregates (the procedure for processing the fluorescence decay curves is described in the Materials and Methods section). An increase of fluorescence lifetime by several orders of magnitude, along with a similar increase in the fluorescence quantum yield of ThT is a characteristic of the dye interaction with amyloid fibrils. An increase in fluorescence lifetime is due to the limited mobility of fragments of the molecular rotor ThT due to the rigidity of the microenvironment (its incorporation into the grooves of amyloid fibrils formed by the side chains of amino acids forming beta-sheets). Thus, the detected increase in the fluorescence lifetime of ThT upon binding to aggregates formed from Vicilin and its fragments by almost 3 orders of magnitude allowed us to confirm the amyloid nature of these aggregates. Additionally, close values of the fluorescence lifetimes of ThT bound to Vicilin, Cupin-1.1, and Cupin-1.2 fibrils indicate a similarity of the dye binding sites, and therefore, a similar structure of amyloid fibers formed from Vicilin and its fragments. Discussion of this question has been added to the manuscript.

**4.      In the aggregation seeding experiments, it was not clear how seeds were produced and why they were defined as 'seeds'.**

- According to the literature, inoculation of amyloid fibrils into fresh solutions of the monomeric protein leads to the formation of replicate fibrils with close structural similarity or identity to the original fibrillar seeds (Scherpelz KP, Lu JX, Tycko R, Meredith SC. Preparation of Amyloid Fibrils Seeded from Brain and Meninges. Methods Mol Biol. 2016;1345:299–312. doi:10.1007/978-1-4939-2978-8_20). In our work seeds have been obtained on the basis of pre-incubated Vicilin, Cupin-1.1 or Cupin-1.2 (protein dissolution in 50% HFIP→evaporation of HFIP→incubation in distilled water). These probes contained amyloid fibrils and non-fibrillar aggregates (in various proportions for different proteins). These seeds were added to the samples of Vicilin, Cupin-1.1 or Cupin-1.2 at the beginning of fibrillogenesis in 1% (V/V) concentration for induction of Vicilin amyloid fibrils formation. Using the Vicilin and Cupin-1.2 seeds allowed us to obtain the *bona fide* Vicilin amyloid fibrils (Fig. 3 a, left and right Panels). A more detailed description of the aggregation seeding experiments was added to the manuscript. Also, we have initially used designation ‘seeds’ to discriminate amyloid seeds from plant seeds.

**5.      The Discussion should be more concise and less repeating the results.**

- We have partially changed Discussion section and incorporated there all issues suggested by Reviewers.

-------

Finally, we would like to thank all Reviewers for very useful comments and interesting suggestions.
