# Supplementary material for: Accumulation of storage proteins in plant seeds is mediated by amyloid formation
Source: PLoS Biol. 2020 Jul 23;18(7):e3000564. doi: 10.1371/journal.pbio.3000564 (PMC7377382; doi:10.1371/journal.pbio.3000564)
Supplement: Attachment — Submitted filename: Response_to_reviewers 2.docx [file pbio.3000564.s018.docx]

**RESPONSE TO THE COMMENTS OF THE REVIEWERS:**

**Rev. 1:**

**The authors have added additional experimental data and significantly strengthened the evidence for the amyloid nature of these protein aggregates. I support their contention that some functional amyloids are dissociated by hot SDS and also agree that not all amyloids are 90% beta sheet. Many functional amyloids retain large non-beta-folded domains in the fibrillar form, where these domains do not contribute to the cross-beta core structure.**

**Points to be addressed.**

**Figure 3. The XRFD of the vicilin fibrils does indeed show the expected cross-beta reflections. However, the samples of Cupin-1.1 and Cupin-1.2 are very salty and the sharp and intense reflections arising from buffer and/or salt in the samples makes it difficult to assign the protein-derived reflections clearly. The authors should collect XRFD data from washed fibrils if possible.**

- Thank you for this comment. We have extended the description of the procedure for X-ray diffraction samples preparation (see lines 1280-1285 (Materials and methods) and lines 514-515 and 544-547 (Results)). To additionally wash obtained fibrils, we have used equilibrium dialysis. Samples in dialysis tubing (Scienova GmbH, Germany) with a pore size of 12-14 kDa were placed into containers with filtered deionized water in the ratio sample/water = 1/500 (v/v). Water was changed after 2 hours, then after next 3 hours and, finally, after a night of incubation of the samples. Dialysis has been finished one day after the start of the incubation. Despite Vicilin samples were almost clear, both Cupin-1.1 and Cupin-1.2 samples exhibited additional reflections indicating presence of salt crystals. These reflections were impossible to be removed by additional washing and dialysis. We suppose that this effect could occur due to salt binding by the fibrils of Cupins.

**In Figure 5, parts j-m showing TEM images - the scale bar is stated to be 200 microns. Is this correct? Perhaps this should be 200 nm? Otherwise the fibrils are inexplicably large.**

- Thank you, it was our mistake. Of course, scale bar should be equal to 200 nm. We have corrected this issue (line 664).

**Rev. 2:**

**All the previous critiques were addressed accordingly. The manuscript reads well in its current state and clarity has increased. This is an important study that will increase the understanding about the biogenesis of amyloids and open a new track in discovery of Plant amyloid proteins.**

- Thank you for this comment.

**Rev. 3:**

**The authors have addressed all my concerns/questions and the manuscript has improved immensely. Small comment: Figure 1(e) is lacking a title and units of the x -axis.**

- Thank you, we have corrected this issue by adding ‘Time, days’ title and units for x-axis.

**__________________________________________________**

**In compliance with data protection regulations, you may request that we remove your personal registration details at any time. (Use the following URL: https://www.editorialmanager.com/pbiology/login.asp?a=r). Please contact the publication office if you have any questions.**

-------

Finally, we would like to thank all Reviewers for very useful comments and interesting suggestions.
